# Integrative physiological, metabolomic, and transcriptomic analysis reveals the drought responses of two apple rootstock cultivars

**DOI:** 10.1186/s12870-024-04902-2

**Published:** 2024-03-26

**Authors:** Xiaohan Li, Yitong Liu, Wei Hu, Baoying Yin, Bowen Liang, Zhongyong Li, Xueying Zhang, Jizhong Xu, Shasha Zhou

**Affiliations:** College of Horticulture, Hebei Agricultural University, Baoding, Hebei 071000 China

**Keywords:** Apple rootstocks, Drought responses, Physiological, Metabolome, Transcriptome

## Abstract

**Background:**

Drought is considered the main environmental factor restricting apple production and thus the development of the apple industry. Rootstocks play an important role in enhancing the drought tolerance of apple plants. Studies of the physiology have demonstrated that ‘ZC9-3’ is a strong drought-resistant rootstock, whereas ‘Jizhen-2’ is a weak drought-resistant rootstock. However, the metabolites in these two apple rootstock varieties that respond to drought stress have not yet been characterized, and the molecular mechanisms underlying their responses to drought stress remain unclear.

**Results:**

In this study, the physiological and molecular mechanisms underlying differences in the drought resistance of ‘Jizhen-2’ (drought-sensitive) and ‘ZC9-3’ (drought-resistant) apple rootstocks were explored. Under drought stress, the relative water content of the leaves was maintained at higher levels in ‘ZC9-3’ than in ‘Jizhen-2’, and the photosynthetic, antioxidant, and osmoregulatory capacities of ‘ZC9-3’ were stronger than those of ‘Jizhen-2’. Metabolome analysis revealed a total of 95 and 156 differentially accumulated metabolites in ‘Jizhen-2’ and ‘ZC9-3’ under drought stress, respectively. The up-regulated metabolites in the two cultivars were mainly amino acids and derivatives. Transcriptome analysis revealed that there were more differentially expressed genes and transcription factors in ‘ZC9-3’ than in ‘Jizhen-2’ throughout the drought treatment. Metabolomic and transcriptomic analysis revealed that amino acid biosynthesis pathways play key roles in mediating drought resistance in apple rootstocks. A total of 13 metabolites, including L-α-aminoadipate, L-homoserine, L-threonine, L-isoleucine, L-valine, L-leucine, (2*S*)-2-isopropylmalate, anthranilate, L-tryptophan, L-phenylalanine, L-tyrosine, L-glutamate, and L-proline, play an important role in the difference in drought resistance between ‘ZC9-3’ and ‘Jizhen-2’. In addition, 13 genes encoding *O*-acetylserine-(thiol)-lyase, *S*-adenosylmethionine synthetase, ketol-acid isomeroreductase, dihydroxyacid dehydratase, isopropylmalate isomerase, branched-chain aminotransferase, pyruvate kinase, 3-dehydroquinate dehydratase/shikimate 5-dehydrogenase, *N*-acetylglutamate-5-P-reductase, and pyrroline-5-carboxylate synthetase positively regulate the response of ‘ZC9-3’ to drought stress.

**Conclusions:**

This study enhances our understanding of the response of apple rootstocks to drought stress at the physiological, metabolic, and transcriptional levels and provides key insights that will aid the cultivation of drought-resistant apple rootstock cultivars. Especially, it identifies key metabolites and genes underlying the drought resistance of apple rootstocks.

**Supplementary Information:**

The online version contains supplementary material available at 10.1186/s12870-024-04902-2.

## Background

Apple (*Malus domestica* Borkh.) is one of the most economically important fruits worldwide, and China is the world’s largest apple producer. Given that most apple trees are grown in arid or semi-arid regions, drought is considered the main environmental factor restricting apple production and thus the development of the apple industry [1, 2]. The study on the yield of ‘Fuji’ under drought stress found that moderate drought stress and severe drought stress resulted in a reduction of 9.34% and 22.94% in yield per plant, respectively [3]. The study on the yield of ‘Naganofuji No.2’ revealed that drought stress led to a decrease in individual plant yield by 26.74% [4]. Drought stress inhibits plant growth through many physiological processes, including chlorophyll biosynthesis, photosynthesis, energy consumption, and reactive oxygen species (ROS) metabolism [5]. The malondialdehyde (MDA) content is used as an indicator of oxidative damage in plants and thus plant stress responses [6]. The main strategies that plants employ to resist drought stress include increasing the activity of antioxidant enzymes, such as superoxide dismutase (SOD), and the accumulation of osmoregulatory substances, such as proline (Pro) [7].

Rootstocks are essential for the grafting and cultivation of fruit trees and play a key role in maintaining the productivity of apple orchards because of their contributions to water and nutrient uptake and abiotic stress resistance [8]. Previous studies have shown that scions on drought-tolerant rootstocks exhibit high stress tolerance, and scions on drought-sensitive rootstocks show reduced stress tolerance [9, 10]. Therefore, in areas with an insufficient water supply, selecting drought-resistant rootstocks for the grafting of apple varieties will enhance the adaptation of apple varieties to water shortages and improve the water conservation, quality, and yield of fruit trees [11, 12]. ‘Jizhen-2’ and ‘ZC9-3’ are two new apple rootstocks cultivated by Hebei Agricultural University. Wang [13] conducted a study of the physiology of these two cultivars and found that ‘ZC9-3’ is a strong drought-resistant rootstock, whereas ‘Jizhen-2’ is a weak drought-resistant rootstock.

The relationship of the drought tolerance of apple rootstocks with the metabolome and transcriptome has received increased attention in light of studies aimed at clarifying the physiological responses of apple rootstocks to drought [5, 9, 14]. The mechanism underlying drought regulation in plants can be clarified using multi-omics joint analysis, and this approach has been widely used in modern biology to characterize the molecular response of plants to abiotic stress [15]. Metabolomics can reveal these complex mechanisms by identifying the metabolites involved in various biochemical processes. The regulation of metabolic networks in plants is altered in response to abiotic stress, and this mediates the synthesis of a series of metabolites that play a role in damage repair [16]. High-throughput transcriptome analysis can be used to identify transcripts with functional information in plant genomes, and this approach has been widely used in plant stress research [17]. Metabolomic and transcriptomic analyses are an effective approach for exploring the responses of plants to drought stress. Yang et al. [18] found that amino acid biosynthetic pathways play important roles in the drought tolerance of two *Haloxylon* species, and Hu et al. [19] identified four amino acid synthesis–related genes (*ASP3*, *ASNS*, *PK*, and *AK*) in *Zanthoxylum bungeanum* leaves under drought stress, which were significantly positively correlated with most of the amino acids.

In this study, to gain insights into the physiological and molecular mechanisms underlying differences in the drought resistance of ‘Jizhen-2’ and ‘ZC9-3’, we conducted a comprehensive study involving physiological, comparative metabolomic and transcriptomic analyses of these two apple rootstock varieties subjected to control conditions (hereafter Jizhen-2-CK and ZC9-3-CK, respectively) and drought conditions (hereafter Jizhen-2-D and ZC9-3-D, respectively). We revealed several key metabolites and genes contributed to the difference in drought resistance between ‘Jizhen-2’ and ‘ZC9-3’. This study provides theoretical support for future studies examining the physiological and molecular mechanisms underlying the responses of apple rootstocks to drought stress, and will aid the cultivation of promising new apple rootstock varieties with drought resistance.

## Materials and methods

### Plant materials and experimental design

The experiments were conducted at Hebei Agricultural University, Baoding, China (38°49’ N, 115°26′ E). At the beginning of April 2020, one-year-old apomictic *Malus hupehensis* (Pamp.) Rehd. *var. mengshanensis* seedlings (bought from Qingdao Academy of Agricultural Sciences, Shandong, China) were planted in plastic pots (the upper diameter, lower diameter, and height of the flowerpot were 30, 20, and 22 cm, respectively) filled with a local clay: fine sand: cow dung mix (3: 1: 1, v: v: v). In mid-April, the buds of two apple rootstocks (‘Jizhen-2’ and ‘ZC9-3’) (sourced from our rootstock resource nursery, in Mancheng District, Baoding City, Hebei Province, China) were grafted onto *Malus hupehensis* (Pamp.) Rehd. *var. mengshanensis* seedlings (60 plants each), and they were subjected to routine management practices until July 31 (day 0). ‘Jizhen-2’ and ‘ZC9-3’ (selected and bred by Apple Science and Technology Innovation Team of Hebei Agricultural University) are the seedling progeny of ‘SH40’ (*Malus pumila* cv. Ralls×*Malus honanensis* Rehd.) and ‘P22’ (one of the P-line cold resistant dwarfing rootstocks selected for breeding in Poland), respectively. On day 1, we selected 50 plants with uniform growth status from each rootstock-scion combination and divided them into two groups for treatment: normal watering (CK, the relative water content of the soil was maintained at 75–85%) and drought stress (D, after the soil was thoroughly watered, irrigation was withheld). Each treatment included 25 plants, and samples (mature functional leaves in the middle of each plant) were taken on day 8 (the relative water content of the soil of Jizhen-2-D and ZC9-3-D was 24–27%) to measure a series of physiological indexes and study the metabolome and transcriptome. Samples were wiped clean, quick-frozen in liquid nitrogen, and then stored in a refrigerator at -80 ℃.

### Determination of physiological indexes

We calculated the weight of the pots when the soil was at 75–85% of the maximum water-holding capacity, and the weight of the pots was measured at 18:00 every day during the treatment period. The moisture lost during the day was replenished based on the difference between weight measurements to maintain a specified soil-moisture content for Jizhen-2-CK and ZC9-3-CK. On day 8, we used the formula [(soil weight − soil dry weight) / (soil saturated weight − soil dry weight)] × 100% to calculate the relative soil moisture content [20]. Following the method described by Liu et al. [9], the relative water content of the leaves (RWC) was calculated using the formula: RWC= [(FM- DM) / (TM-DM)] × 100%, where FM, TM, and DM represent the fresh, turgid, and dry mass of the leaves, respectively. Chlorophyll *a* (Chl a), chlorophyll *b* (Chl b), total chlorophyll (Chl t), and carotenoids (Car) were extracted from the leaves and estimated according to the method suggested by Arnon [21]. Photosynthetic measurements were determined using a Li-Cor 6400 portable photosynthesis system (Li-6400XT, LICOR, Lincoln, Nebraska, USA). The net photosynthetic rate (Pn), stomatal conductance (Gs), intercellular CO_2_ concentration (Ci), and transpiration rate (Tr) were measured from mature leaves between 09:00 AM and 11:00 AM on sunny days. Instantaneous water use efficiency (WUE_I_) was calculated by dividing Pn by Tr [22]; both parameters were measured simultaneously at a stable photosynthetically active radiation (PAR). The superoxide anion (${O}_{2}^{\stackrel{-}{\bullet }}$) production rate, malondialdehyde (MDA) content, superoxide dismutase (SOD) activity, and proline (Pro) content were determined following the methods described by Ru et al. [23]. Three biological replicates were carried out for each sample in the above experiments.

### Metabolite profiling using UPLC-MS/MS

According to the standard procedures of Wuhan MetWare Biotechnology Co., Ltd. (www.metware.cn), sample preparation, extract analysis, and metabolite identification and quantification were performed.

For each sample (Jizhen-2-CK, Jizhen-2-D, ZC9-3-CK, and ZC9-3-D), three biological replicates were independently analyzed. Extraction of metabolites were performed following the methods described by Xue et al. [24], before ultra-performance liquid chromatography (UPLC)-tandem mass spectrometry (MS/MS) analysis. UPLC and MS/MS were performed using SHIMADZU Nexera X2 (www.shimadzu.com.cn/) and Applied Biosystems 4500 Q TRAP (www.appliedbiosystems.com.cn/), respectively, chromatographic pure metabolite samples as standard samples. UPLC conditions and electrospray ionization-quadrupole-linear ion trap-MS/MS (ESI-Q TRAP-MS/MS) were as described by Mei et al. [25]. Qualitative and quantitative determination of metabolites was performed based on the methods in Yang et al. [26]. Based on the self-built database MWDB (metware database), secondary mass spectrometry data were subjected to qualitative analysis. Interference from isotope signals; duplicate signals of K^+^, Na^+^, and NH4^+^ ions; and duplicate signals of fragment ions derived from other relatively large molecules were excluded during qualitative analysis. Quantitative analysis of metabolites was performed by the multiple reaction monitoring (MRM) mode of the triple quadrupole (QQQ) mass spectrometry. After obtaining the metabolite mass spectrometry data of the different samples, peak area integration was performed on all mass spectrum peaks, and the mass spectral peaks of the same metabolite in different samples were corrected. The chromatographic peak area was used to determine the relative metabolite content.

### Metabolome data analysis

The accumulation pattern of metabolites among different samples was analyzed by hierarchical cluster analysis (HCA) using R software (www.r-project.org/). The filtered data were submitted to Simca-P software (version 13.0, Umetrics AB, Umea, Sweden) for unsupervised principal component analysis (PCA) and orthogonal partial least squares-discriminant analysis (OPLS-DA). Differentially accumulated metabolites (DAMs) were identified using the following criteria: fold change (FC) ≥ 2 or ≤ 0.5 and variable importance in projection (VIP) ≥ 1. VIP values were extracted from the OPLS-DA results. Identified metabolites were annotated using the Kyoto Encyclopedia of Genes and Genomes (KEGG) Compound database (http://www.kegg.jp/kegg/compound/); annotated metabolites were then mapped to the KEGG Pathway database (http://www.kegg.jp/kegg/pathway.html). Pathways with significantly regulated metabolites mapped were then subjected to metabolite set enrichment analysis, and the significance of these pathways was determined according to the *p*-values of hypergeometric tests.

### RNA‑Seq analysis

Extraction of total RNA and construction of cDNA library were performed following the methods described by Li et al. [27]. Sequencing and quality control of cDNA library were carried out according to Meng et al. [28]. Alignment analysis between the clean reads and the reference genomes was conducted using HISAT2 [29].

Feature Counts software, FPKM (Fragments Per Kilobase of transcript per Million mapped reads), and DESeq2 software were used for gene counting, measuring gene expression levels, and analyzing the differentially expressed genes (DEGs) between different comparison groups, respectively [24]. The DEGs were identified using the following criteria: |log2FC| ≥ 1 and false discovery rate (FDR) < 0.05. FC represents the ratio of gene expression values in two groups. The resulting *P*-values were adjusted using the Benjamini and Hochberg’s FDR [30]. The functions of unigenes were annotated by seven public databases, including KEGG, Gene Ontology (GO), eukaryotic Ortholog Groups (KOG), NCBI non-redundant protein sequences (Nr), Protein family (Pfam), a manually annotated and curated protein sequence database (Swiss-Prot), and Trembl [27]. KOBAS software was used to test the statistical enrichment of DEGs in KEGG pathways [31]. GO analysis of the DEGs was performed using the GOSeq R package (corrected *p*-values < 0.05) [32].

### Quantitative real-time PCR (qRT-PCR) analysis

Ten genes were subjected to qRT-PCR to verify the RNA-Seq results. Total RNA was extracted from the leaves of Jizhen-2-CK, Jizhen-2-D, ZC9-3-CK, and ZC9-3-D using the Tiangen RNA Pure Plant kit (Tiangen, Beijing, China). The concentration, purity, and integrity of total RNA were measured using a NanoDrop 2000 spectrophotometer (Thermo Scientific, Wilmington, DE, USA). The synthesis of first-strand cDNA was performed using the TransScript® One-Step gDNA Removal and cDNA Synthesis SuperMix (TransGen Biotech Co., Ltd., Beijing, China). The qRT-PCR reaction was performed using the TransStart® Top Green qPCR SuperMix (TransGen Biotech Co., Ltd., Beijing, China) and LightCycler® 96 Real-Time PCR System (Qiangxin Biorepublic Co., Ltd, Beijing, China). All the expression level data obtained through qRT-PCR were based on three biological replicates. The relative gene expression levels were calculated using the 2^−ΔΔCT^ method. The *MdActin* gene was used to normalize gene expression levels, and the gene-specific primers in Table S1 were designed using Primer 6.0.

### KEGG pathway analysis of the differentially accumulated metabolites and differentially expressed genes

Using the DAMs and DEGs in response to drought stress from one of the two apple rootstock varieties as data, and mapping them to KEGG database simultaneously, metabolic pathways and KEGG pathway annotation diagrams involving differential metabolites and genes can be obtained. KEGG pathway annotation diagrams can display the accumulation of metabolites, the expression of genes encoding different enzymes, and the EC numbers of enzymes.

### Statistical analysis

The data were analyzed using one-way analysis of variance (ANOVA) and Duncan’s multiple range test using IBM SPSS 25.0 Statistics (SPSS Inc., Chicago, IL, USA); the threshold for statistical significance was *P* < 0.05. Correlation analysis of 12 samples was performed using BMKCloud (https://www.biocloud.net). Graphs were drawn using Origin 2021 (OriginLab, Northampton, Massachusetts, USA). The numbers shown were generated by Microsoft Excel 2021.

## Results

### Effect of drought stress on the phenotypes and physiological indexes of ‘Jizhen-2’ and ‘ZC9-3’

**Fig. 1:**
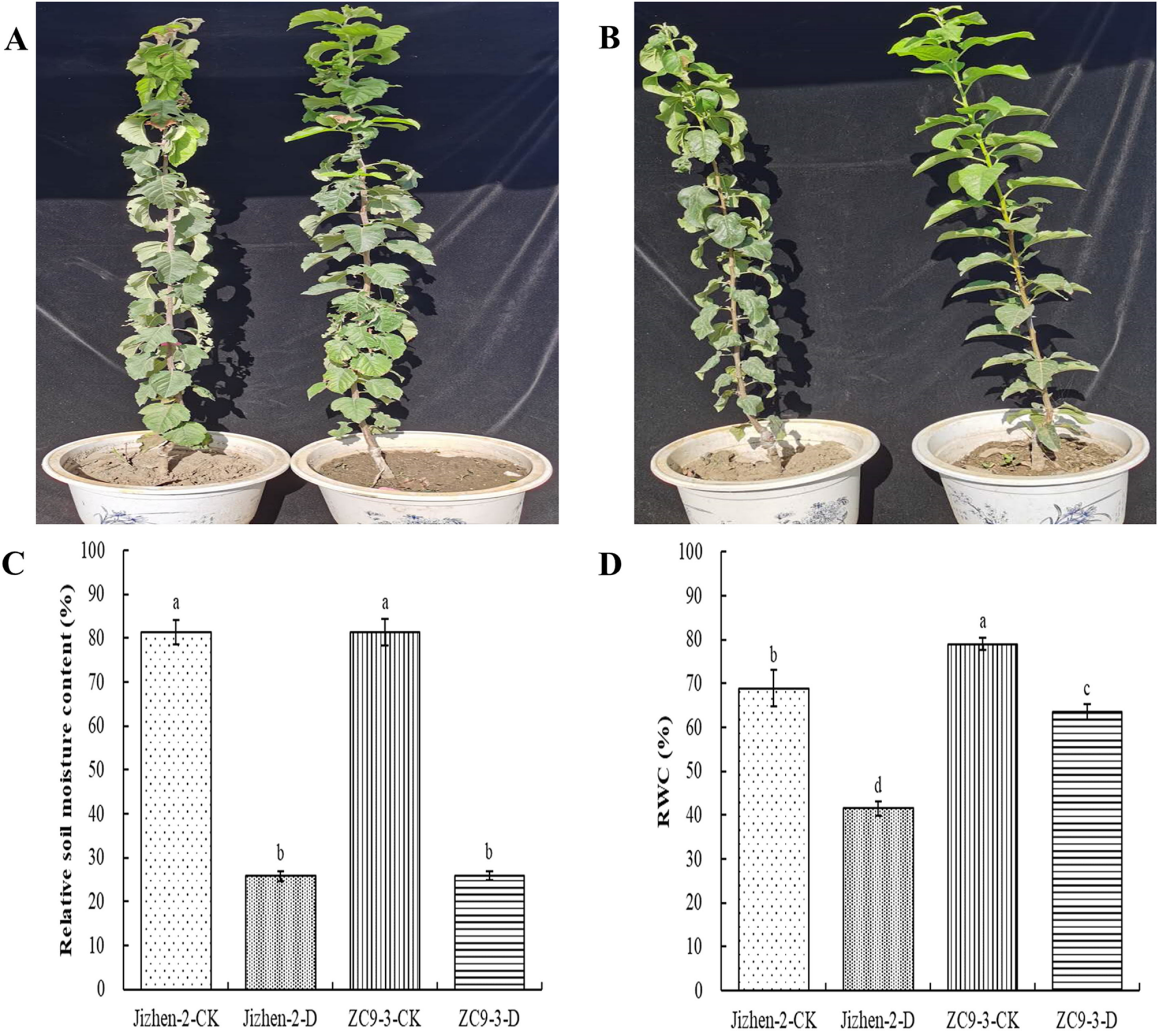
Effect of drought stress on the phenotypes and physiological indicators of ‘Jizhen-2’ and ‘ZC9-3’. (**A**), Phenotypes of Jizhen-2-D (left) and Jizhen-2-CK (right). (**B**), Phenotypes of ZC9-3-D (left) and ZC9-3-CK (right). (**C**), Relative soil moisture content (%). (**D**), Leaf relative water content (RWC, %). CK and D indicate normal watering and drought stress, respectively. Within each panel, bars labeled with different lowercase letters denote significant differences (*P* ≤ 0.05) according to Duncan’s tests. Each point represents the mean value from three biological replicates, and the vertical bars indicate standard deviations (SDs). The same applies to subsequent figures

Differences in the wilting degree, curling state, and scorching degree of the leaves under normal watering and drought stress conditions were more significant in ‘Jizhen-2’ than in ‘ZC9-3’ (Fig. 1A-B). The relative soil moisture content and RWC of these two apple rootstock varieties decreased significantly under drought stress (Fig. 1C-D). The relative soil moisture content of Jizhen-2-CK and ZC9-3-CK was between 75% and 85%, and the relative soil moisture content of Jizhen-2-D and ZC9-3-D was between 24% and 27%. The percent difference in RWC of ‘Jizhen-2’ and ‘ZC9-3’ between drought and normal watering conditions was − 39.74% and − 19.45%, respectively.

Drought stress significantly increased the content of Chl a, Chl b, and Car in the leaves of ‘Jizhen-2’ and ‘ZC9-3’ (Table 1). The increase in the above three indexes was higher in ‘ZC9-3’ than in ‘Jizhen-2’. The Pn, Tr, Ci, and Gs were significantly lower, and WUE_I_ was significantly higher in these two apple rootstock varieties under drought stress compared with under normal watering conditions (Table 2). The decline in the Pn, Tr, Ci, and Gs of ‘Jizhen-2’ was greater than that of ‘ZC9-3’, and the increase in WUE_I_ in ‘ZC9-3’ was greater than that in ‘Jizhen-2’.

**Fig. 2:**
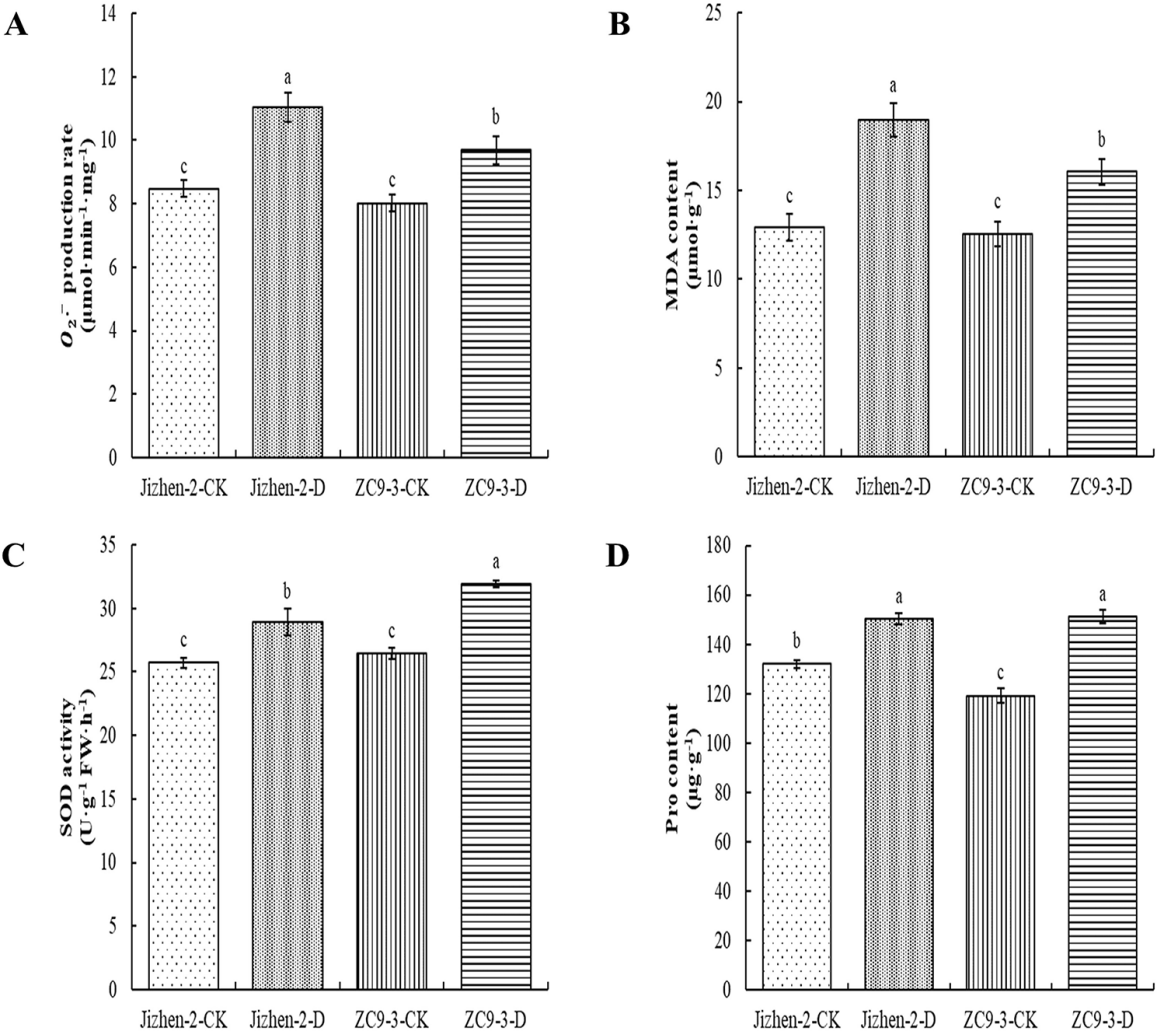
Effect of drought stress on the superoxide anion (${O}_{2}^{\stackrel{-}{\bullet }}$) production rate (**A**), malondialdehyde (MDA) content (**B**), superoxide dismutase (SOD) activity (**C**), and proline (Pro) content (**D**) of ‘Jizhen-2’ and ‘ZC9-3’

Drought stress resulted in a significant increase in the ${O}_{2}^{\stackrel{-}{\bullet }}$ production rate and MDA content in the leaves of these two apple rootstock varieties (Fig. 2A-B). The ${O}_{2}^{\stackrel{-}{\bullet }}$ production rate was 30.41% and 20.79% higher in ‘Jizhen-2’ and ‘ZC9-3’ under drought stress compared with under normal watering conditions, respectively. The MDA content of ‘Jizhen-2’ and ‘ZC9-3’ was 47.08% and 27.79% higher under drought stress compared with under normal watering conditions, respectively. The SOD activity and Pro content in the leaves of these two apple rootstock varieties were significantly higher under drought stress compared with under normal watering conditions (Fig. 2C-D). The SOD activity of ‘Jizhen-2’ and ‘ZC9-3’ was 12.38% and 20.70% higher and the Pro content was 13.82% and 26.88% higher under drought stress compared with under normal watering conditions, respectively.

**Table 1:** Effect of drought stress on the content of photosynthetic pigments of ‘Jizhen-2’ and ‘ZC9-3’

Photosynthetic pigment content (mg·g^− 1^)	Jizhen-2		Percent difference (%)	ZC9-3		Percent difference (%)
	CK	D		CK	D	
Chl a	1.80 ± 0.03 b	2.07 ± 0.06 a	15.36	1.41 ± 0.02 c	1.84 ± 0.04 b	30.65
Chl b	0.63 ± 0.03 b	0.81 ± 0.03 a	29.50	0.48 ± 0.03 c	0.66 ± 0.02 b	37.01
Chl t	2.42 ± 0.05 b	2.89 ± 0.08 a	19.01	1.89 ± 0.03 c	2.50 ± 0.06 b	32.29
Car	0.34 ± 0.01 b	0.37 ± 0.02 a	8.43	0.25 ± 0.01 c	0.37 ± 0.01 a	47.47

Note: in this table, Chl a, Chl b, Chl t, and Car indicate chlorophyll *a*, chlorophyll *b*, total chlorophyll, and carotenoids, respectively. Data are presented as the mean values ± SDs of three biological replicates. Different lowercase letters within a column indicate significant differences at *P* ≤ 0.05 according to Duncan’s tests; the same applies to subsequent tables

**Table 2:** Effect of drought stress on the photosynthetic parameters of ‘Jizhen-2’ and ‘ZC9-3’

Photosynthetic parameters	Jizhen-2		Percent difference (%)	ZC9-3		Percent difference (%)
	CK	D		CK	D	
Pn (µmol·m^− 2^·s^− 1^)	10.82 ± 0.21 a	2.31 ± 0.20 c	-78.62	11.42 ± 0.61 a	4.73 ± 0.18 b	-58.61
Tr (mmol·m^− 2^·s^− 1^)	5.76 ± 0.11 a	0.62 ± 0.01 c	-89.21	5.86 ± 0.03 a	1.01 ± 0.07 b	-82.79
Ci (µmol·mol^− 1^)	325.54 ± 4.15 a	247.39 ± 5.53 c	-24.00	321.03 ± 1.78 a	260.46 ± 2.95 b	-18.87
Gs (mol·m^− 2^·s^− 1^)	0.288 ± 0.010 a	0.016 ± 0.001 b	-94.44	0.295 ± 0.008 a	0.024 ± 0.001 b	-91.93
WUE_I_ (µmol·mmol^− 1^)	1.88 ± 0.07 c	3.73 ± 0.32 b	98.08	1.95 ± 0.10 c	4.38 ± 0.17 a	124.60

Note: Pn, Tr, Ci, Gs, and WUE_I_ indicate net photosynthetic rate, transpiration rate, intracellular CO_2_ concentration, stomatal conductance and instantaneous water use efficiency, respectively

### Effect of drought stress on the metabolome of ‘Jizhen-2’ and ‘ZC9-3’

#### Data quality assessment

A total of 693 metabolites were detected in samples from Jizhen-2-CK, Jizhen-2-D, ZC9-3-CK, and ZC9-3-D, and each sample comprised three biological replicates (Table S2, Fig. S1). To ensure the repeatability and reliability of the data, the overlap ratios of the MS results of the different quality control (QC) samples were calculated (Fig. S2). The high overlap ratio of the total ion current curves of the QC samples indicated that the results were reliable.

#### Overview of the metabolites of ‘Jizhen-2’ and ‘ZC9-3’

**Fig. 3:**
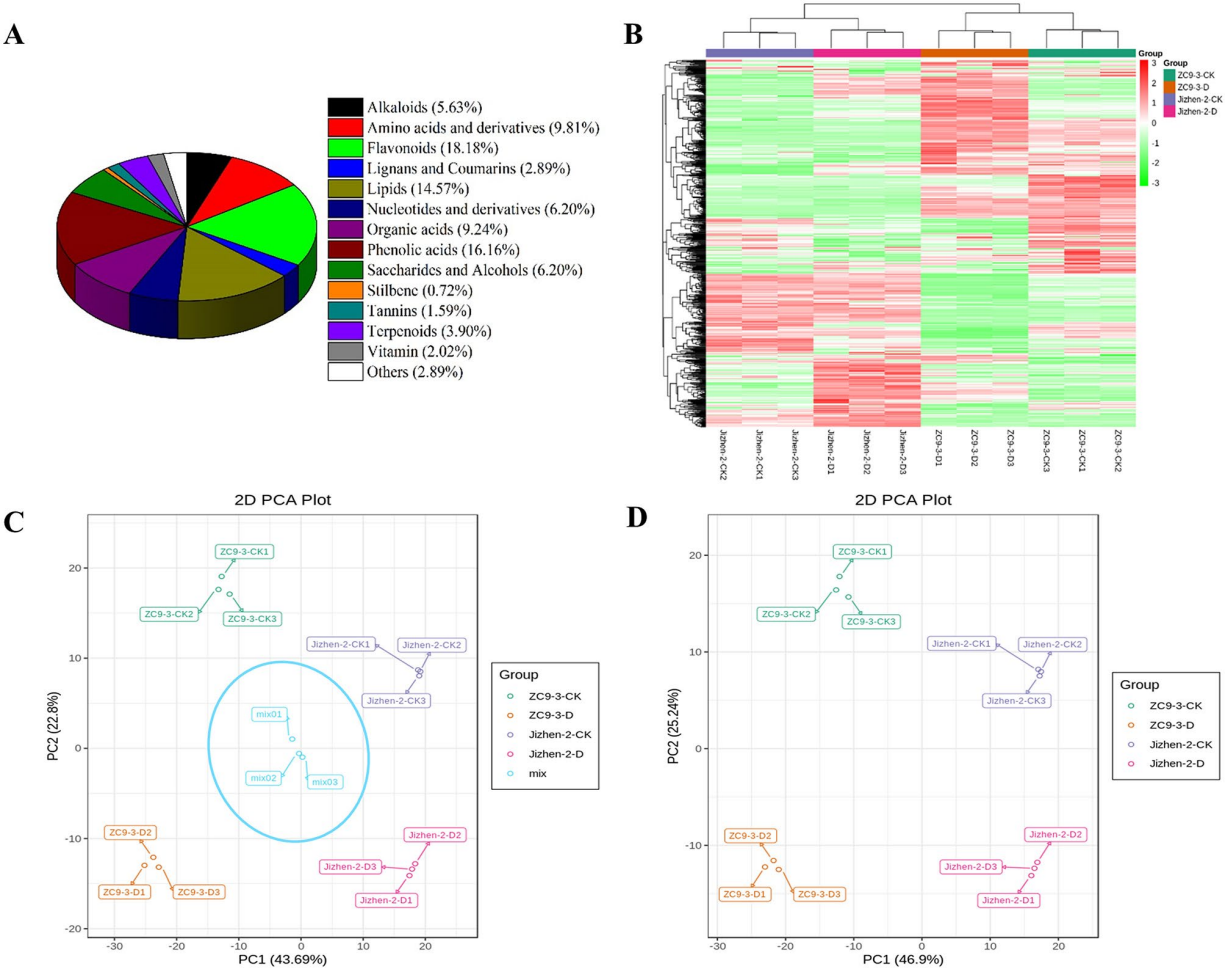
Qualitative and quantitative analysis of the metabolomics data for ‘Jizhen-2’ and ‘ZC9-3’. (**A**), Classification of the 693 metabolites. (**B**), Heatmap of the quantified metabolites. (**C**), Principal component analysis (PCA) of 12 samples and QC samples (mix indicates QC samples). (**D**), PCA of Jizhen-2-CK, Jizhen-2-D, ZC9-3-CK, and ZC9-3-D samples

A total of 693 metabolites were detected in the four groups of samples (Fig. 3A), and these could be divided into 14 classes, including 39 alkaloids, 68 amino acids and derivatives, 126 flavonoids, 20 lignans and coumarins, 101 lipids, 43 nucleotides and derivatives, 64 organic acids, 112 phenolic acids, 43 saccharides and alcohols, 5 stilbenes, 11 tannins, 27 terpenoids, 14 vitamins, and 20 others. The accumulation patterns of metabolites among apple rootstock samples were analyzed by HCA, and the results are shown in a heatmap (Fig. 3B).

The heatmap revealed that changes in the content of several metabolites in the leaves under drought stress and normal watering conditions differed in ‘Jizhen-2’ and ‘ZC9-3’, indicating that the metabolic processes affected by drought stress in ‘Jizhen-2’ may be different from those affected by drought stress in ‘ZC9-3’. In addition, the three biological replicates in each group of samples were clustered together, indicating there was a high degree of similarity among replicates and thus that the data were reliable. The results of a PCA of all treatments and QC samples are shown in Fig. 3C. Variation within each group was low, and the three duplicate QC samples were clustered in the center of the PCA plot, indicating that the data were highly repeatable and reliable. In the PCA of the four groups of samples (Fig. 3D), the first principal component was correlated with differences in different apple rootstock varieties and explained 46.90% of the variance in the data; the second principal component was correlated with differences among treatments and explained 25.24% of the variation in the data. Furthermore, the replicate samples were clustered, indicating that the results of the experiment were repeatable and reliable; the metabolites in the leaves of ‘Jizhen-2’ and ‘ZC9-3’ changed significantly after drought stress, and this was consistent with observed changes in physiological indicators.

#### Differential accumulation of drought-resistant metabolites in ‘Jizhen-2’ and ‘ZC9-3’

**Fig. 4:**
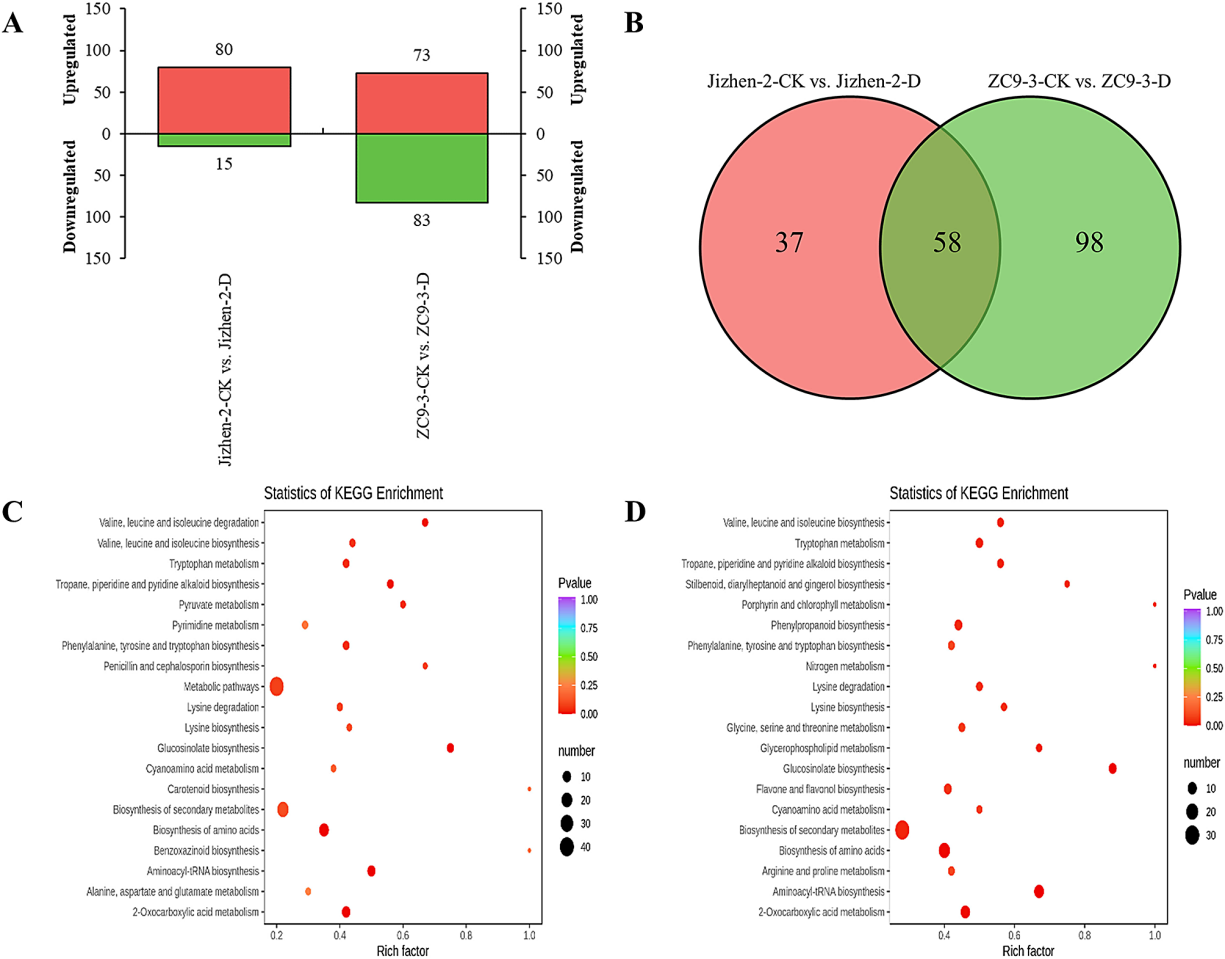
Metabolome analysis of ‘Jizhen-2’ and ‘ZC9-3’ under drought and normal watering conditions. (**A**), The number of DAMs in the Jizhen-2-CK vs. Jizhen-2-D and ZC9-3-CK vs. ZC9-3-D comparisons. (**B**), Venn diagram of DAMs in the Jizhen-2-CK vs. Jizhen-2-D and ZC9-3-CK vs. ZC9-3-D comparisons. (**C**), Pathway enrichment analysis of DAMs in the Jizhen-2-CK vs. Jizhen-2-D comparison. (**D**), Pathway enrichment analysis of DAMs in the ZC9-3-CK vs. ZC9-3-D comparison

Based on the OPLS-DA results, the number of DAMs with significant differences were determined for the two groups (Jizhen-2-CK vs. Jizhen-2-D and ZC9-3-CK vs. ZC9-3-D). A total of 95 DAMs were identified in the Jizhen-2-CK vs. Jizhen-2-D comparison (Fig. 4A), and the number of up-regulated metabolites was greater than the number of down-regulated metabolites (Table S3). A total of 156 DAMs were detected in the ZC9-3-CK vs. ZC9-3-D comparison, and the number of up-regulated metabolites was less than the number of down-regulated metabolites (Table S4). Table 3 shows the numbers of up-regulated and down-regulated DAMs in ‘Jizhen-2’ and ‘ZC9-3’ under drought stress and normal watering conditions. The DAMs in the Jizhen-2-CK vs. Jizhen-2-D comparison belonged to 11 classes, and most DAMs belonged to five classes, including amino acids and derivatives (22.11%), lipids (17.89%), alkaloids (13.68%), organic acids (12.63%), and flavonoids (10.53%). The DAMs in the ZC9-3-CK vs. ZC9-3-D comparison could be divided into 13 classes. Phenolic acids (21.79%), flavonoids (19.87%), amino acids and derivatives (14.74%), and alkaloids (12.82%) accounted for a large proportion of the DAMs in this comparison. We also found that up-regulated amino acids and derivatives were the most common up-regulated DAMs in these two apple rootstock varieties. The total content of amino acids and derivatives in the two cultivars was higher under drought stress than under normal watering, and the magnitude of the increase was greater in ‘ZC9-3’ than in ‘Jizhen-2’. A Venn diagram was used to clarify the DAMs specific to each pairwise comparison, as well as those shared among the two pairwise comparisons. More than half of the DAMs (58 metabolites) in ‘Jizhen-2’ were shared; however, less than half of the DAMs in ‘ZC9-3’ were shared (Fig. 4B). Furthermore, most of the shared DAMs were amino acids and derivatives (31.03%), and the observed changes in these DAMs in the two pairwise comparisons were similar (17 up-regulated and 1 down-regulated) (Table S5). Overall, our results indicate that amino acids and derivatives were the major DAMs in ‘Jizhen-2’ and ‘ZC9-3’ under drought stress; these were thus considered to be the key metabolites mediating the response of these two cultivars to drought stress.

The enriched metabolic pathways from the KEGG pathway enrichment analysis are shown in Fig. 4C-D. In the Jizhen-2-CK vs. Jizhen-2-D comparison, the DAMs were mainly enriched in 2-oxocarboxylic acid metabolism, aminoacyl-tRNA biosynthesis, biosynthesis of amino acids, biosynthesis of secondary metabolites, and metabolic pathways. In the ZC9-3-CK vs. ZC9-3-D comparison, the DAMs were mainly enriched in 2-oxocarboxylic acid metabolism, aminoacyl-tRNA biosynthesis, biosynthesis of amino acids, biosynthesis of secondary metabolites, flavone and flavonol biosynthesis, and phenylpropanoid biosynthesis.

**Table 3:** Numbers of up-regulated and down-regulated DAMs in different metabolite classes in ‘Jizhen-2’ and ‘ZC9-3’ under drought and normal watering conditions

Class	Jizhen-2-CK vs. Jizhen-2-D			ZC9-3-CK vs. ZC9-3-D	
	Up	Down		Up	Down
Alkaloids	12	1		15	5
Amino acids and derivatives	20	1		20	3
Flavonoids	4	6		7	24
Lignans and Coumarins	4	1		1	4
Lipids	17	0		9	0
Nucleotides and derivatives	3	2		4	1
Organic acids	9	3		5	4
Phenolic acids	3	0		4	30
Saccharides and Alcohols	5	1		6	0
Stilbene	0	0		0	1
Terpenoids	0	0		0	8
Vitamin	2	0		0	1
Others	1	0		2	2
Total	80	15		73	83

### Effect of drought stress on the transcriptome of ‘Jizhen-2’ and ‘ZC9-3’

#### RNA‑Seq analysis of the leaves of ‘Jizhen-2’ and ‘ZC9-3’

To explore the molecular mechanism underlying the effects of drought stress on the two apple rootstock varieties, we collected the leaves of Jizhen-2-CK, Jizhen-2-D, ZC9-3-CK, and ZC9-3-D, and each sample in the transcriptome analysis comprised three biological replicates. After filtering the original data, the sequencing error rate and GC content distribution of the 12 transcriptome samples were determined, and the number of clean reads for subsequent analysis were obtained. The 12 transcriptome samples yielded 78.52 Gb of clean data, with at least 6.00 Gb of clean data for each transcriptome sample. In each transcriptome sample, the percentage of Q30 bases and GC content exceeded 93.10% and 46.71%, respectively, indicating that the quality of the transcriptome sequencing data was relatively high. The clean reads were mapped to the apple genome, with the mapping ratio ranging from 89.75 to 91.05% (Table S6).

#### Differential expression analysis of ‘Jizhen-2’ and ‘ZC9-3’

**Fig. 5:**
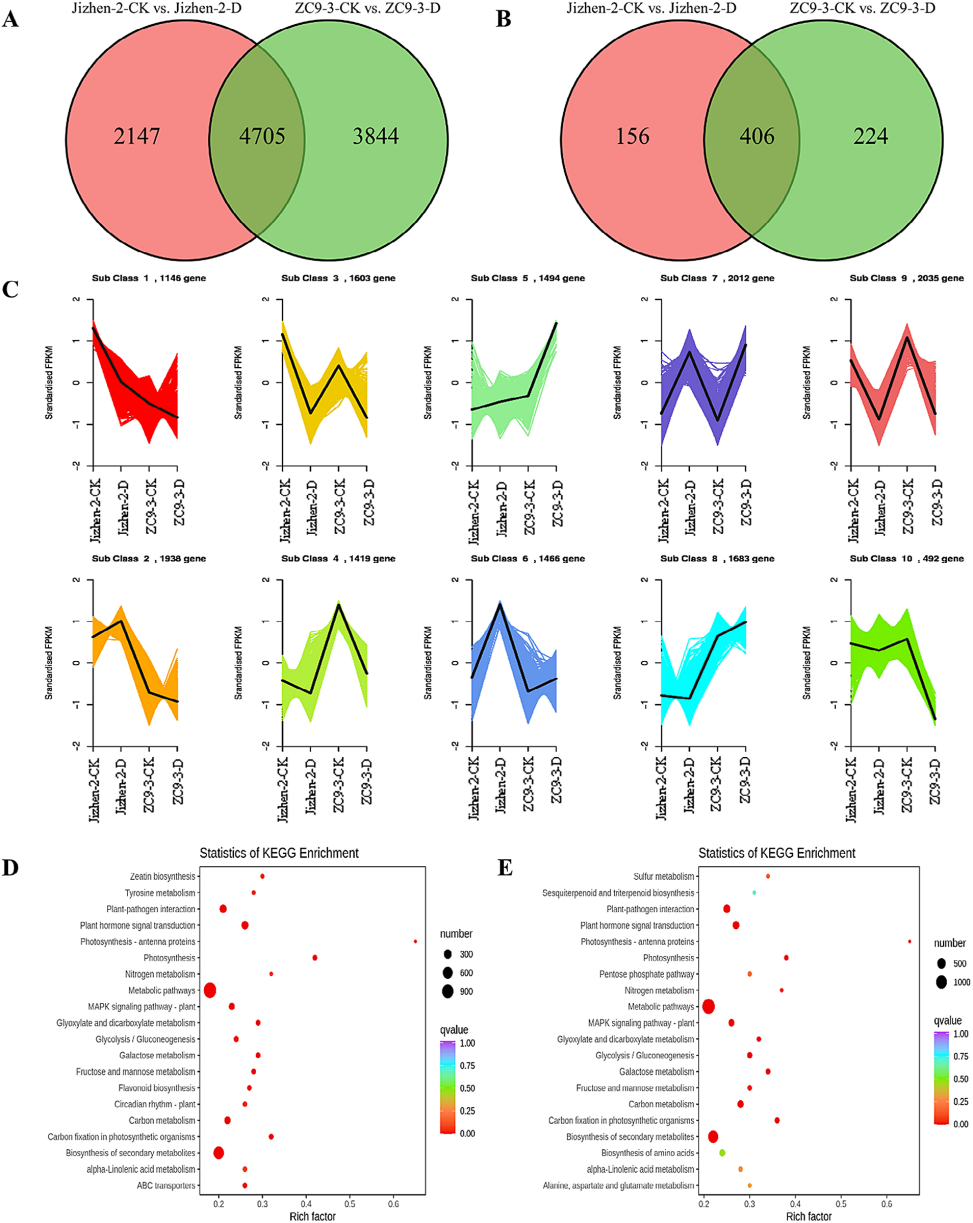
Transcriptome analysis of ‘Jizhen-2’ and ‘ZC9-3’ under drought and normal watering conditions. (**A**), Venn diagram of DEGs in the Jizhen-2-CK vs. Jizhen-2-D and ZC9-3-CK vs. ZC9-3-D comparisons. (**B**), Venn diagram of differentially expressed transcription factors (TFs) in the Jizhen-2-CK vs. Jizhen-2-D and ZC9-3-CK vs. ZC9-3-D comparisons. (**C**), K-means clustering analysis of gene expression patterns in Jizhen-2-CK, Jizhen-2-D, ZC9-3-CK, and ZC9-3-D. (**D**), Pathway enrichment analysis of DEGs in the Jizhen-2-CK vs. Jizhen-2-D comparison. (**E**), Pathway enrichment analysis of DEGs in the ZC9-3-CK vs. ZC9-3-D comparison

In the Jizhen-2-CK vs. Jizhen-2-D and ZC9-3-CK vs. ZC9-3-D comparisons, 6,852 (2,568 up-regulated, 4,284 down-regulated) DEGs and 8,549 (2,994 up-regulated, 5,555 down-regulated) DEGs were identified (Fig. S3), respectively. There were 4,705 common DEGs and 406 common differentially expressed transcription factors (TFs) in the two pairwise comparisons (Fig. 5A-B).

To analyze DEGs expression patterns, the expression data of Jizhen-2-CK, Jizhen-2-D, ZC9-3-CK, and ZC9-3-D were centralized and standardized and then clustered using K-means. The DEGs in the four groups of samples were clustered into 10 subclasses (Fig. 5C). The DEGs in subclasses 2, 7, and 9 were significantly enriched in the four groups of samples. The DEGs in subclasses 5 and 7 were up-regulated to a greater degree in the ZC9-3-CK vs. ZC9-3-D comparison than in the Jizhen-2-CK vs. Jizhen-2-D comparison. The DEGs in subclasses 6 were up-regulated to a greater degree in the Jizhen-2-CK vs. Jizhen-2-D comparison than in the ZC9-3-CK vs. ZC9-3-D comparison. The DEGs in subclasses 4, 9, and 10 were down-regulated to a greater degree in the ZC9-3-CK vs. ZC9-3-D comparison than in the Jizhen-2-CK vs. Jizhen-2-D comparison. The DEGs in subclasses 1 and 3 were down-regulated to a greater degree in the Jizhen-2-CK vs. Jizhen-2-D comparison than in the ZC9-3-CK vs. ZC9-3-D comparison. The DEGs in subclasses 2 were up-regulated in the Jizhen-2-CK vs. Jizhen-2-D comparison and down-regulated in the ZC9-3-CK vs. ZC9-3-D comparison. The DEGs in subclasses 8 were up-regulated in the ZC9-3-CK vs. ZC9-3-D comparison and down-regulated in the Jizhen-2-CK vs. Jizhen-2-D comparison.

We performed GO enrichment analysis of the above-mentioned DEGs and obtained significantly enriched GO terms for the DEGs (Fig. S4). The GO terms for DEGs in the Jizhen-2-CK vs. Jizhen-2-D and ZC9-3-CK vs. ZC9-3-D comparisons were compared; the DEGs for the two pairwise comparisons were significantly enriched in cellular process (GO:0009987) and metabolic process (GO:0008152) in the biological process category. Cell (GO:0005623), cell part (GO:0044464), organelle (GO:0043226), and membrane (GO:0016020) were the main enriched GO terms in the cellular component category. In the molecular function category, the main GO terms were binding (GO:0005488) and catalytic activity (GO:0003824). To further clarify the biological functions of DEGs, we analyzed significantly enriched pathways to identify key metabolic pathways and signal pathways (Table S7-S8, Fig. 5D-E). The most highly enriched pathways for DEGs in the Jizhen-2-CK vs. Jizhen-2-D and ZC9-3-CK vs. ZC9-3-D comparisons were metabolic pathways (1,164 DEGs and 1,393 DEGs, respectively), biosynthesis of secondary metabolites (712 DEGs and 816 DEGs, respectively), plant-pathogen interaction (214 DEGs and 262 DEGs, respectively), plant hormone signal transduction (212 DEGs and 222 DEGs, respectively) and carbon metabolism (148 DEGs and 189 DEGs, respectively). In addition, the DEGs in the ZC9-3-CK vs. ZC9-3-D comparison were significantly enriched in biosynthesis of amino acids. In conclusion, the analysis of DEGs revealed that the difference in the drought resistance of ‘Jizhen-2’ and ‘ZC9-3’ might stem from the roles of DEGs in these metabolic processes.

**Fig. 6:**
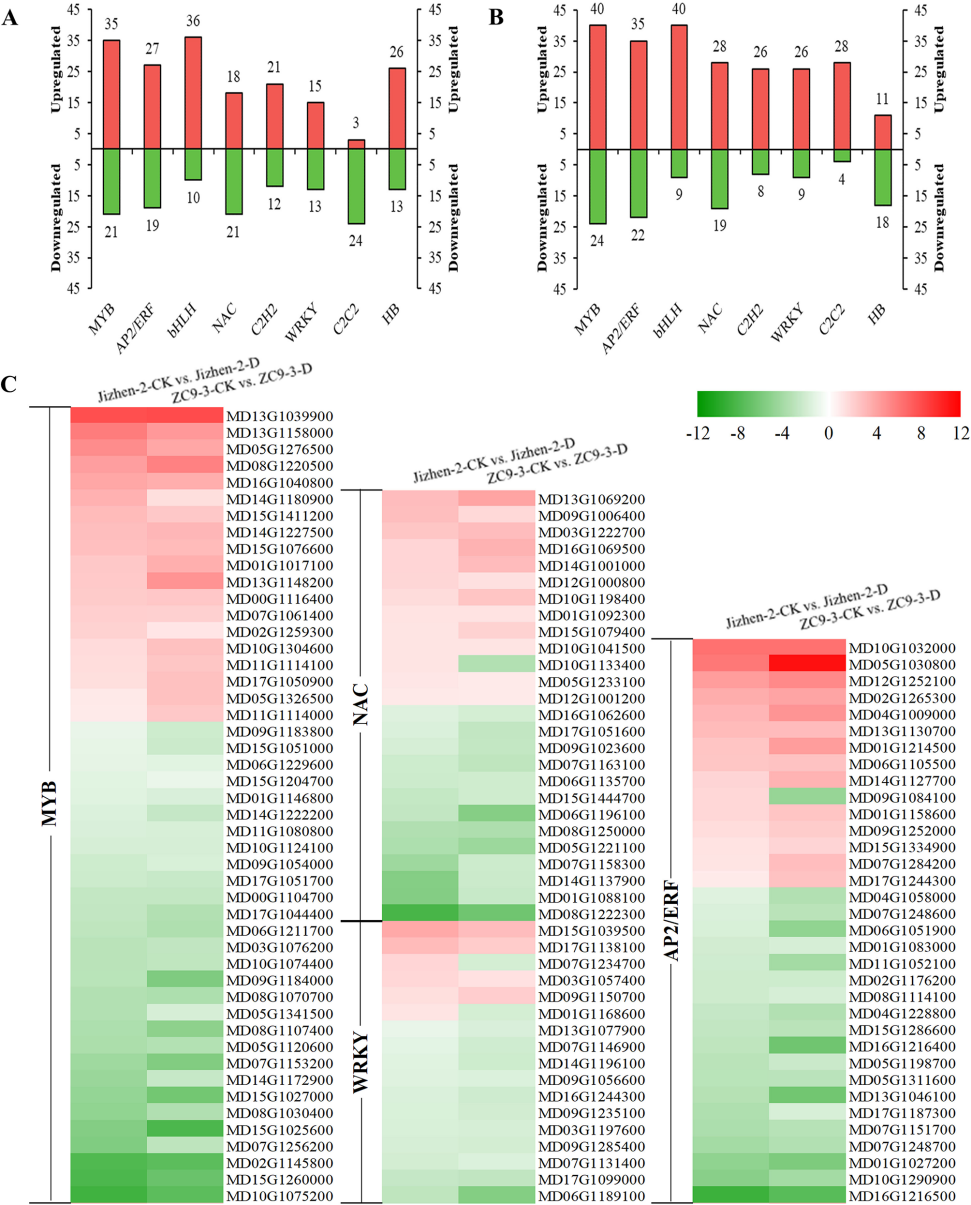
Analysis of differentially expressed transcription factors (TFs) of ‘Jizhen-2’ and ‘ZC9-3’. (**A**), The number of up-regulated and down-regulated TFs in various gene families in the Jizhen-2-CK vs. Jizhen-2-D comparison. (**B**), The number of up-regulated and down-regulated TFs in various gene families in the ZC9-3-CK vs. ZC9-3-D comparison. (**C**), Heatmap of the common differentially expressed TFs of MYB, AP2/ERF, NAC, and WRKY families in the two pairwise comparisons, which shows the fold changes in expression (Log2FC) of the genes encoding TFs

TFs, also known as *trans*-acting factors, regulate plant growth and development and environmental stress responses by activating or inhibiting the expression of genes [33]. A total of 562 differentially expressed TFs (197 up-regulated, 365 down-regulated) from 54 gene families were identified in the Jizhen-2-CK vs. Jizhen-2-D comparison (Table S9). A total of 630 differentially expressed TFs (209 up-regulated, 421 down-regulated) from 55 gene families were identified in the ZC9-3-CK vs. ZC9-3-D comparison (Table S10). These differentially expressed TFs in the two varieties mainly belonged to eight gene families, including the MYB, AP2/ERF, bHLH, NAC, C2H2, WRKY, C2C2, and HB families, and the number of differentially expressed TFs in the first seven of these gene families was higher in ‘ZC9-3’ than in ‘Jizhen-2’ (Fig. 6A-B). Numerous studies have shown that four TF families (MYB, AP2/ERF, NAC, and WRKY) play an important role in the response of plants to drought, temperature, salinity, and other types of abiotic stress [34–39]. Analysis of the TFs of these four families revealed that drought stress resulted in consistent changes in the expression of most of the common differentially expressed TFs in the leaves of these two apple rootstock varieties, with the exception of NAC (*MD10G1133400*), WRKY (*MD07G1234700* and *MD01G1168600*), and AP2/ERF (*MD09G1084100*), which were significantly up-regulated in ‘Jizhen-2’ but significantly down-regulated in ‘ZC9-3’ (Fig. 6C).

### Validation of the accuracy of the transcriptomic data

**Fig. 7:**
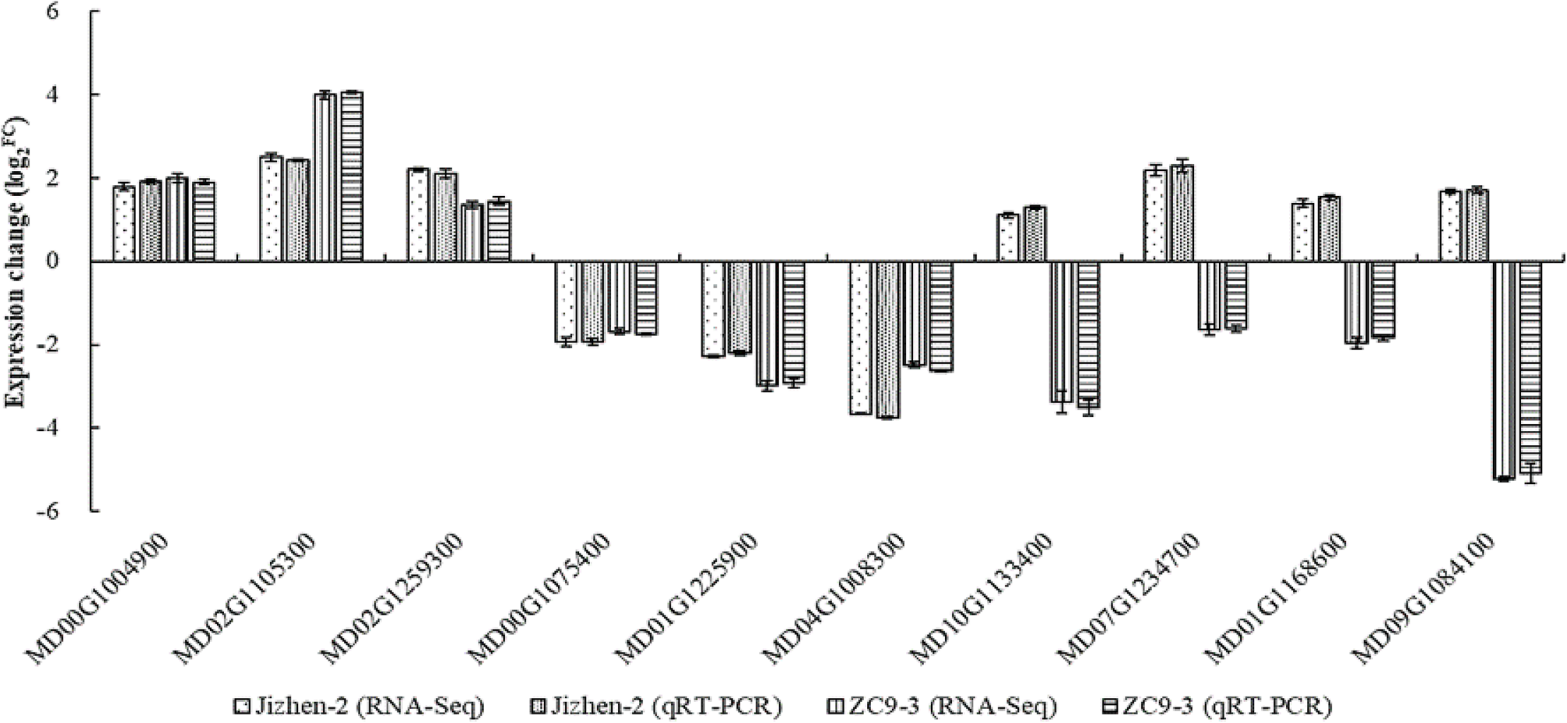
Analysis of qRT-PCR of the expression changes of 10 common DEGs in ‘Jizhen-2’ and ‘ZC9-3’. *MD00G1004900* [transcription factor TCP19-like (LOC103439698)], *MD02G1105300* [alpha-aminoadipic semialdehyde synthase (LOC103454158), transcript variant X2], and *MD02G1259300* [transcription factor DIVARICATA-like (LOC103426918)] are three co-up-regulated DEGs. *MD00G1075400* [glucan endo-1,3-beta-glucosidase 14 (LOC103453353)], *MD01G1225900* [metal-nicotianamine transporter YSL3-like (LOC103444222)], and *MD04G1008300* [MYB family transcription factor APL (MYBR7)] are three co-down-regulated DEGs. *MD10G1133400* [NAC domain-containing protein 22-like (LOC103454651)], *MD07G1234700* [WRKY DNA-binding transcription factor 70 (LOC103432930)], *MD01G1168600* [WRKY DNA-binding transcription factor 70-like (LOC103450764)], and *MD09G1084100* [AP2/ERF and B3 domain-containing transcription repressor RAV2-like (LOC103442871)] are four common differentially expressed TFs

To verify the accuracy of the transcriptome results, we detected the expression of 10 common DEGs in the two cultivars, including three co-up-regulated DEGs, three co-down-regulated DEGs, and four common differentially expressed TFs, using qRT-PCR method. Changes in the expression of the 10 DEGs measured by qRT-PCR were consistent with the RNA-Seq data (Fig. 7), indicating that the transcriptome results were reliable.

### Integrated metabolomic and transcriptomic analysis of ‘Jizhen-2’ and ‘ZC9-3’

A PCA of the transcriptomic and metabolomic data from ‘Jizhen-2’ and ‘ZC9-3’ under different treatments revealed significant differences among the four groups of samples (Fig. 3D and Fig. S5). KEGG enrichment analysis of DAMs and DEGs in the Jizhen-2-CK vs. Jizhen-2-D and ZC9-3-CK vs. ZC9-3-D comparisons revealed the following significantly enriched pathways: metabolic pathways (Jizhen-2-CK vs. Jizhen-2-D: 1,164 DEGs and 41 DAMs; ZC9-3-CK vs. ZC9-3-D: 1,393 DEGs and 48 DAMs), biosynthesis of secondary metabolites (Jizhen-2-CK vs. Jizhen-2-D: 712 DEGs and 23 DAMs; ZC9-3-CK vs. ZC9-3-D: 816 DEGs and 30 DAMs), and biosynthesis of amino acids (Jizhen-2-CK vs. Jizhen-2-D: 117 DEGs and 15 DAMs; ZC9-3-CK vs. ZC9-3-D: 151 DEGs and 17 DAMs) (Fig. S6). Pearson’s correlation coefficients (PCCs) of the DAMs and DEGs in each pairwise comparison were calculated using the ‘Cor’ package in R (https://www.rproject.org/), and the difference multiples of the metabolites with PCC > 0.8 in each group are shown in a nine-quadrant diagram (Fig. S7). A total of 7,711 genes and 99 metabolites in the Jizhen-2-CK vs. Jizhen-2-D comparison were detected in the three and seven quadrants. A total of 9,937 genes and 163 metabolites in the ZC9-3-CK vs. ZC9-3-D comparison were detected in the three and seven quadrants. The differential patterns of gene expression and metabolites were consistent, suggesting that the metabolite changes might be positively regulated by genes. In addition, differential correlation analysis (PCC > 0.8) revealed that 6,426 DEGs were related to 95 DAMs in the Jizhen-2-CK vs. Jizhen-2-D comparison and 8,084 DEGs were associated with 156 DAMs in the ZC9-3-CK vs. ZC9-3-D comparison (Fig. S8).

The two-way orthogonal partial least squares (O2PLS) model was established using all DAMs and DEGs, and variables with high correlations and weights in different datasets were initially evaluated based on the loading plot; important variables affecting the other omics dataset were identified. The 10 metabolites affected most significantly by the transcriptome included five amino acids (L-phenylalanine, L-valine, L-isoleucine, L-norleucine, and L-leucine), four alkaloids (4,5,6-trihydroxy-2-cyclohexen-1-ylideneacetonitrile, piperidine, 6-deoxyfagomine, and *N*-benzylmethylene isomethylamine), and one nucleotide derivative (6-methylmercaptopurine) (Fig. S9). Moreover, the 30 genes most significantly affected by the metabolome are shown in Table S11. The expression levels of most of these genes (29 genes) were higher in Jizhen-2-D than in the other samples, and the expression levels of two genes (*MD16G1165100* and *MD05G1136400*) were high in all samples. The expression level of one gene (*MD08G1237700*) was higher in ZC9-3-CK and ZC9-3-D samples than in Jizhen-2-CK and Jizhen-2-D samples.

### Amino acid biosynthesis pathway of ‘Jizhen-2’ and ‘ZC9-3’ in response to drought stress

**Fig. 8:**
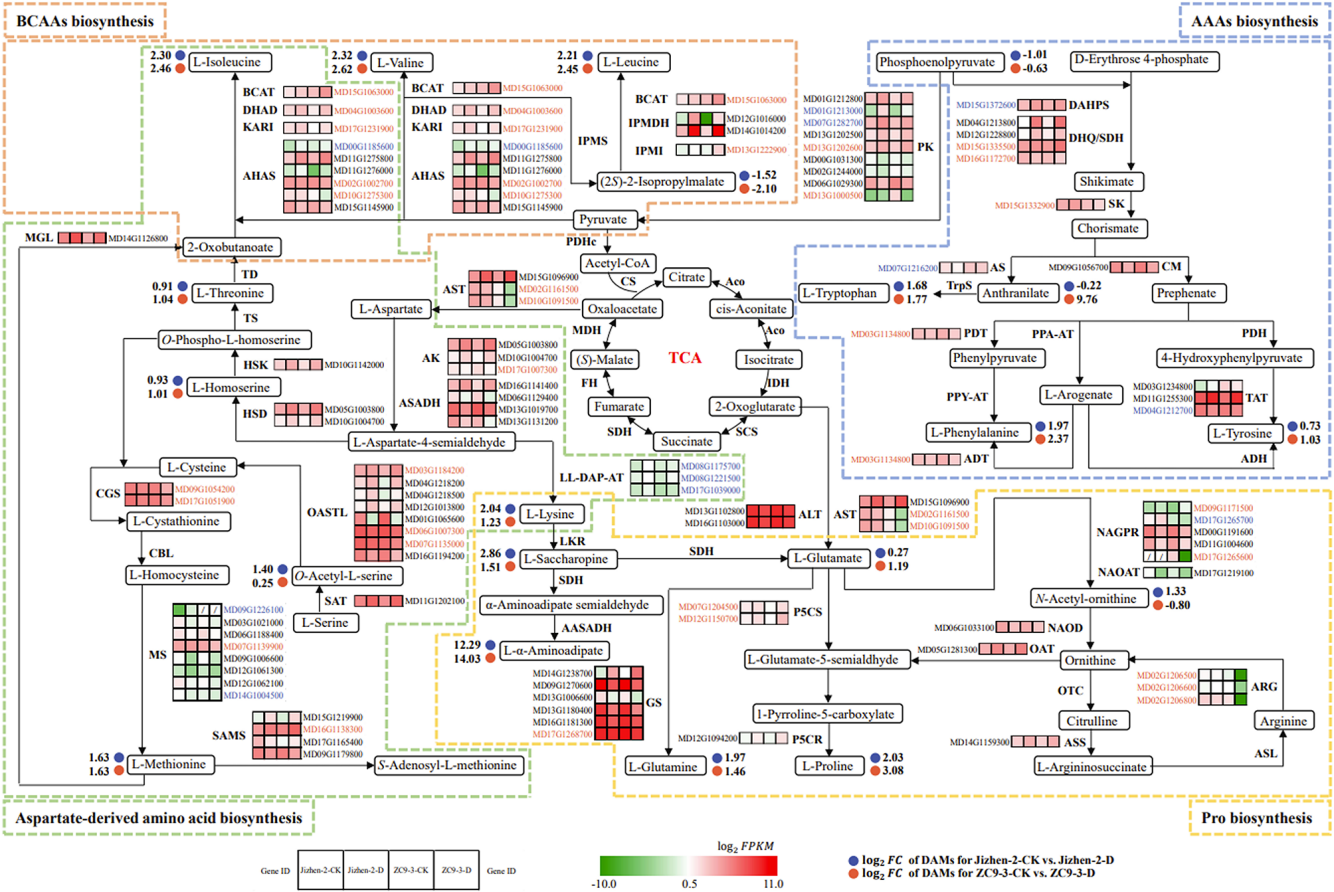
Amino acid biosynthesis pathway in ‘Jizhen-2’ and ‘ZC9-3’ under drought stress and normal watering conditions

Our metabolomic analysis revealed that amino acids and derivatives were the major DAMs in ‘Jizhen-2’ and ‘ZC9-3’; they are thus considered to be the key metabolites mediating the drought resistance of the two apple rootstock varieties. The DAMs in the two cultivars were mainly enriched in the amino acid biosynthesis pathway. In the transcriptomic analysis, the DEGs in ‘ZC9-3’, but not ‘Jizhen-2’, were significantly enriched in the amino acid biosynthesis pathway. In the comprehensive metabolomic and transcriptomic analysis, the DAMs and DEGs in ‘Jizhen-2’ and ‘ZC9-3’ were significantly enriched in the amino acid biosynthesis pathway. Hence, we focused on the amino acid biosynthesis pathway (Fig. 8).

Lysine, methionine, threonine and isoleucine are commonly referred to as aspartate-derived amino acids because they are synthesized from aspartate through a branched pathway [40]. Isoleucine, valine and leucine are collectively referred to as branched-chain amino acids (BCAAs) because of their short branched hydrophobic side chains [41]. In the aspartate-derived amino acid pathway and BCAAs biosynthesis pathway, the ${\text{log}}_{2}FC$ values of L-lysine and (2*S*)-2-isopropylmalate were higher in the Jizhen-2-CK vs. Jizhen-2-D comparison than in the ZC9-3-CK vs. ZC9-3-D comparison, but the opposite pattern was observed for the ${\text{log}}_{2}FC$ values of BCAAs. After drought stress, *O*-acetyl-L-serine was significantly up-regulated only in ‘Jizhen-2’, and L-homoserine and L-threonine were significantly up-regulated only in ‘ZC9-3’. The magnitude of changes in the expression of most common DEGs (13 co-up-regulated DEGs and 6 co-down-regulated DEGs) was smaller in the Jizhen-2-CK vs. Jizhen-2-D comparison than in the ZC9-3-CK vs. ZC9-3-D comparison. After drought stress, one *S*-adenosylmethionine synthetase (SAMS) gene (*MD17G1165400*) was significantly down-regulated and up-regulated in ‘Jizhen-2’ and ‘ZC9-3’, respectively. Five genes were significantly up-regulated only in ‘Jizhen-2’, including three LL-diaminopimelate aminotransferase (LL-DAP-AT) genes (*MD08G1175700*, *MD08G1221500*, and *MD17G1039000*), one methionine synthase (MS) gene (*MD09G1226100*), and one acetohydroxyacid synthase (AHAS) gene (*MD00G1185600*). One MS gene (*MD14G1004500*) was significantly down-regulated only in ‘Jizhen-2’. Six genes were significantly up-regulated only in ‘ZC9-3’, including one *O*-acetylserine-(thiol)-lyase (OASTL) gene (*MD03G1184200*), one SAMS gene (*MD16G1138300*), one ketol-acid isomeroreductase (KARI) gene (*MD17G1231900*), one dihydroxyacid dehydratase (DHAD) gene (*MD04G1003600*), one isopropylmalate isomerase (IPMI) gene (*MD13G1222900*), and one branched-chain aminotransferase (BCAT) gene (*MD15G1063000*). Ten genes were significantly down-regulated only in ‘ZC9-3’, including two aspartate aminotransferase (AST) genes (*MD02G1161500* and *MD10G1091500*), one aspartate kinase (AK) gene (*MD17G1007300*), two OASTL genes (*MD06G1007300* and *MD07G1135000*), two cystathionine γ-synthase (CGS) genes (*MD09G1054200* and *MD17G1051900*), one MS gene (*MD07G1139900*), and two AHAS genes (*MD02G1002700* and *MD10G1275300*).

Tryptophan, phenylalanine, and tyrosine are collectively referred to as aromatic amino acids (AAAs). In the AAAs biosynthesis pathway, we found that the ${\text{log}}_{2}FC$ values of L-phenylalanine and L-tryptophan were lower in the Jizhen-2-CK vs. Jizhen-2-D comparison than in the ZC9-3-CK vs. ZC9-3-D comparison. After drought stress, phosphoenolpyruvate was significantly down-regulated only in ‘Jizhen-2’, and anthranilate and L-tyrosine were significantly up-regulated only in ‘ZC9-3’. The expression changes of two-thirds of the common DEGs (four co-up-regulated DEGs and two co-down-regulated DEGs) were higher in the Jizhen-2-CK vs. Jizhen-2-D comparison than in the ZC9-3-CK vs. ZC9-3-D comparison. After drought stress, one tyrosine aminotransferase (TAT) gene (*MD03G1234800*) was significantly up-regulated in ‘Jizhen-2’ but significantly down-regulated in ‘ZC9-3’. Moreover, two pyruvate kinase (PK) genes (*MD01G1213000* and *MD07G1282700*) and one 3-deoxy-d-arabino-heptulosonate-7-phosphate synthase (DAHPS) gene (*MD15G1372600*) were significantly up-regulated only in ‘Jizhen-2’. One anthranilate synthase (AS) gene (*MD07G1216200*) and one TAT gene (*MD04G1212700*) were significantly down-regulated only in ‘Jizhen-2’. One PK gene (*MD13G1202600*) and one 3-dehydroquinate dehydratase/shikimate 5-dehydrogenase (DHQ/SDH) gene (*MD15G1335500*) were significantly up-regulated only in ‘ZC9-3’. One PK gene (*MD13G1000500*), one DHQ/SDH gene (*MD16G1172700*), one shikimate kinase (SK) gene (*MD15G1332900*), one prephenate dehydratase (PDT) gene (*MD03G1134800*), and one arogenate dehydratase (ADT) gene (*MD03G1134800*) were significantly down-regulated only in ‘ZC9-3’.

In plants, Pro can be synthesized from glutamate and ornithine through a common intermediate, glutamate-5-semialdehyde [42]. In the Pro biosynthesis pathway, the ${\text{log}}_{2}FC$ values of L-α-aminoadipate and L-Pro were lower in the Jizhen-2-CK vs. Jizhen-2-D comparison than in the ZC9-3-CK vs. ZC9-3-D comparison, but the opposite pattern was observed for the ${\text{log}}_{2}FC$ values of L-saccharopine and L-glutamine. After drought stress, *N*-acetyl-ornithine was significantly up-regulated only in ‘Jizhen-2’, and L-glutamate was significantly up-regulated only in ‘ZC9-3’. We also found that the expression changes of two-thirds of the common DEGs (five co-up-regulated DEGs and five co-down-regulated DEGs) were lower in the Jizhen-2-CK vs. Jizhen-2-D comparison than in the ZC9-3-CK vs. ZC9-3-D comparison. After drought stress, one *N*-acetylglutamate-5-P-reductase (NAGPR) gene (*MD17G1265700*) was significantly up-regulated only in ‘Jizhen-2’. Three genes were significantly up-regulated only in ‘ZC9-3’, including one NAGPR gene (*MD09G1171500*) and two pyrroline-5-carboxylate synthetase (P5CS) genes (*MD07G1204500* and *MD12G1150700*). Seven genes were significantly down-regulated only in ‘ZC9-3’, including two AST genes (*MD02G1161500* and *MD10G1091500*), one NAGPR gene (*MD17G1265600*), three arginase (ARG) genes (*MD02G1206500*, *MD02G1206600*, and *MD02G1206800*), and one glutamine synthetase (GS) gene (*MD17G1268700*).

Based on the above results, some metabolites, such as L-α-aminoadipate, L-homoserine, L-threonine, BCAAs, (2*S*)-2-isopropylmalate, anthranilate, AAAs, L-glutamate, and L-Pro, might contribute to the difference in drought resistance of ‘Jizhen-2’ and ‘ZC9-3’. After drought stress, opposite expression patterns of one SAMS gene (*MD17G1165400*) and one TAT gene (*MD03G1234800*) were observed in ‘Jizhen-2’ and ‘ZC9-3’, and this is related to differences in drought resistance between these two apple rootstock varieties. Some genes were significantly up-regulated only in ‘ZC9-3’, and these genes encoded OASTL, SAMS, KARI, DHAD, IPMI, BCAT, PK, DHQ/SDH, NAGPR, and P5CS, which play a positive role in regulating the response of ‘ZC9-3’ to drought stress.

The heatmap was drawn using log_2_FPKM. Red and green indicate high expression and low expression, respectively. Blue and orange gene IDs indicate the specific DEGs in ‘Jizhen-2’ and ‘ZC9-3’, respectively. Black gene IDs indicate the common DEGs in ‘Jizhen-2’ and ‘ZC9-3’. The abbreviations and EC numbers of the enzymes in the network map are shown in Table S12.

## Discussion

Drought stress is one of the main factors limiting apple cultivation, and rootstocks play an important role in enhancing the drought tolerance of apple plants [14]. There is thus a need to develop rootstocks with enhanced drought resistance. The decrease in RWC induced by drought stress was less pronounced in ‘ZC9-3’ than in ‘Jizhen-2’, indicating that the water retention capacity of the leaves of ‘ZC9-3’ is higher than that of ‘Jizhen-2’. The decrease in RWC might have stemmed from the lack of water in the soil or root systems, which are not able to compensate for the water lost by transpiration because of a reduction in the absorbing surface area [43]. Photosynthetic pigments, especially chlorophyll, play a key role in plant photosynthesis, and the chlorophyll content often declines under drought conditions [1, 9, 44]. However, we found that drought stress significantly increased the Chl a and Chl b content of ‘Jizhen-2’ and ‘ZC9-3’ on day 8, which is consistent with the results of Sik et al. [45], indicating that the photosynthetic capacity of the chloroplast is not substantially damaged by drought conditions. Under drought stress, the normal photosynthesis of plants is inhibited by stomatal and non-stomatal limitations. Similar patterns of changes in Gs and Ci indicate inhibition caused by stomatal limitation; however, contrasting patterns of changes in Gs and Ci indicate inhibition caused by non-stomatal limitation [46]. Drought stress led to a significant decrease in the Pn, Tr, Ci, and Gs of ‘Jizhen-2’ and ‘ZC9-3’, indicating that stomatal limitation was the cause of the decrease in leaf photosynthesis under drought stress. In addition, decreases in the Pn, Tr, Ci, and Gs were more pronounced in ‘Jizhen-2’ than in ‘ZC9-3’, and the increase in WUE_I_ was more pronounced in ‘ZC9-3’ than in ‘Jizhen-2’, indicating that photosynthesis was not as strongly inhibited by drought in ‘ZC9-3’ compared with ‘Jizhen-2’.

One of the main effects of drought stress on plants is the excessive accumulation of ROS, including hydrogen peroxide (H_2_O_2_) and ${O}_{2}^{\stackrel{-}{\bullet }}$. The massive accumulation of ROS can disrupt the stability of protein, sugar, and nucleic acid molecules and lead to membrane lipid peroxidation, the degree of which can be inferred by the content of MDA [47]. Moreover, ROS detoxification can be achieved by enzymatic and non-enzymatic antioxidants. SOD is an enzymatic antioxidant that plays an important role in the antioxidant defense system by scavenging ${O}_{2}^{\stackrel{-}{\bullet }}$. Car are some of the most common non-enzymatic antioxidants [48]. The MDA content and the production rate of ${O}_{2}^{\stackrel{-}{\bullet }}$ of ‘Jizhen-2’ and ‘ZC9-3’ significantly increased after drought stress, indicating that drought stress induced the production of ROS in cells, which resulted in oxidative reactions and the production of membrane lipid peroxidation products. Increases in the MDA content and ${O}_{2}^{\stackrel{-}{\bullet }}$ production rate were lower in ‘ZC9-3’ than in ‘Jizhen-2’, and increases in the SOD activity and the content of Car were higher in ‘ZC9-3’ than in ‘Jizhen-2’, indicating that the antioxidant capacity of ‘ZC9-3’ is higher than that of ‘Jizhen-2’, which reduces the accumulation of reactive oxygen free radicals. Pro plays an important role in osmotic regulation and protects proteins and cellular membranes from osmotic and oxidative stress [48]. Previous studies have shown that drought stress can promote the synthesis of Pro in leaves, and the Pro content is higher in varieties with strong drought resistance than in varieties with weak drought resistance [49, 50]. In our study, the Pro content was significantly increased in the leaves of ‘Jizhen-2’ and ‘ZC9-3’ following exposure to drought stress, and the increase in the Pro content was less pronounced in ‘Jizhen-2’ than in ‘ZC9-3’, indicating that the osmoregulatory capacity of ‘ZC9-3’ is greater than that of ‘Jizhen-2’. In sum, we suspect that the higher drought resistance of ‘ZC9-3’ compared with ‘Jizhen-2’ stems from the fact that the RWC and photosynthetic, antioxidant, and osmoregulatory capacities of ‘ZC9-3’ are higher than those of ‘Jizhen-2’ under drought stress, which is consistent with the results of a previous study [13].

To further explore the molecular mechanisms underlying differences in drought resistance between ‘Jizhen-2’ and ‘ZC9-3’, we used metabolomic and transcriptomic analyses to analyze the metabolites and genes involved in the drought responses of these two apple rootstock varieties. We identified 13 key metabolites, including L-α-aminoadipate, L-homoserine, L-threonine, BCAAs, (2*S*)-2-isopropylmalate, anthranilate, AAAs, L-glutamate, and L-Pro, as well as 13 key genes, including one OASTL gene (*MD03G1184200*), two SAMS genes (*MD17G1165400* and *MD16G1138300*), one KARI gene (*MD17G1231900*), one DHAD gene (*MD04G1003600*), one IPMI gene (*MD13G1222900*), one BCAT gene (*MD15G1063000*), one PK gene (*MD13G1202600*), one DHQ/SDH gene (*MD15G1335500*), one TAT gene (*MD03G1234800*), one NAGPR gene (*MD09G1171500*), and two P5CS genes (*MD07G1204500* and *MD12G1150700*), that were enriched in the amino acid biosynthesis pathway and contributed to the enhanced drought resistance of ‘ZC9-3’ compared with that of ‘Jizhen-2’. Amino acid biosynthesis pathway plays a crucial role in plants response to drought stress. The metabolomic study of two alfalfa cultivars with contrasting tolerance to drought revealed that under drought stress, the DAMs of drought-tolerant alfalfa cultivar were mainly enriched in the amino acid biosynthesis pathway [51]. The metabolomic analysis of the root-tips of drought-tolerant rice showed that under drought stress, the DAMs were mainly enriched in pathways such as amino acid biosynthesis and aminoacyl-tRNA biosynthesis [52]. In addition, the transcriptomic analysis of two maize inbred lines with contrasting drought tolerance revealed that under drought stress, the DEGs in the drought-tolerant maize inbred line were primarily associated with nitrogen metabolism and amino acid biosynthesis pathways [53]. L-Homoserine is a crucial precursor for L-threonine and L-methionine biosynthesis [54]. Because methionine and threonine are used as substrates for isoleucine biosynthesis, their biosynthesis is related to isoleucine availability under abiotic stress [55]. We found that L-homoserine and L-threonine were significantly up-regulated after drought stress only in ‘ZC9-3’. OASTL catalyzes the formation of cysteine using sulfide and the carbon backbone provided by *O*-acetylserine, which is central to the sulfur assimilation process in plants, and it is generally regarded as a non-limiting enzyme without a regulatory function because of its low substrate affinities and semi-constitutive expression patterns [56, 57]. SAMS uses methionine and ATP to generate *S*-adenosyl-L-methionine, which plays an important role in the response to a variety of abiotic stresses [58–60]. In this study, drought stress resulted in the significant up-regulation of one OASTL gene (*MD03G1184200*) and one SAMS gene (*MD16G1138300*) only in ‘ZC9-3’, and one SAMS gene (*MD17G1165400*) was significantly down-regulated and up-regulated in ‘Jizhen-2’ and ‘ZC9-3’, respectively. BCAAs can accumulate in large quantities in plants in response to drought stress, and this might enhance the drought tolerance of plants, given that BCAAs could serve as compatible osmolytes or alternative energy sources [41, 61, 62]. The increase in the content of BCAAs following drought stress was less pronounced in ‘Jizhen-2’ compared with ‘ZC9-3’. This is consistent with the results of previous studies showing that the content of BCAAs is higher in drought-tolerant varieties than in drought-sensitive varieties [63, 64]. A unique feature of BCAA biosynthesis is that isoleucine and valine are synthesized in two parallel pathways. This is achieved with a single set of four enzymes (AHAS, KARI, DHAD, and BCAT), which catalyze a series of reactions that lead to the formation of these amino acids with different substrates [65]. DHAD has been purified from spinach and contains an iron-sulfur cluster [2Fe-2S] [66]. IPMI catalyzes the isomerization of (*S*)-2-isopropylmalate to (2*R*,3*S*)-3-isopropylmalat and is a member of the aconitase (ACN) superfamily, which usually comprises monomeric proteins with [4Fe-4S] clusters. Similar to other Fe–S cluster proteins, DHAD and IPMI are also sensitive to ROS [67, 68]. We found that the content of (2*S*)-2-isopropylmalate was 0.35 times higher in Jizhen-2-D than in Jizhen-2-CK, and the content of (2*S*)-2-isopropylmalate was 0.23 times higher in ZC9-3-D than in ZC9-3-CK. One KARI gene (*MD17G1231900*), one DHAD gene (*MD04G1003600*), one IPMI gene (*MD13G1222900*), and one BCAT gene (*MD15G1063000*) were significantly up-regulated only in ‘ZC9-3’.

In plants, AAAs play key roles in the synthesis of proteins and a wide range of secondary metabolites such as pigments, alkaloids, hormones, and cell wall components, which are important for human nutrition as well as for plant development and growth [69, 70]. Moreover, high levels of AAAs and their related secondary metabolites are associated with high resistance to biotic and abiotic stress in plants [71, 72]. We found that L-tyrosine was significantly up-regulated only in ‘ZC9-3’ under drought stress. The content of L-tryptophan and L-phenylalanine was 3.20 and 3.91 times higher in Jizhen-2-D than in Jizhen-2-CK, respectively. The content of L-tryptophan and L-phenylalanine was 3.40 and 5.18 times higher in ZC9-3-D than in ZC9-3-CK, respectively. PK catalyzes the irreversible transfer of phosphate from phosphoenolpyruvate to ADP, which results in the production of pyruvate and ATP; it thus plays a key role in controlling metabolic flux and ATP production [73]. We found that one PK gene (*MD13G1202600*) was significantly up-regulated only in ‘ZC9-3’ under drought stress. In plants, AAAs are synthesized through the shikimate pathway. The shikimate pathway is initiated by combining phosphoenolpyruvate and erythrose-4-phosphate with the activity of DAHPS, and then undergoes five enzymatic reactions that are sequentially catalyzed by 3-dehydroquinate synthase (DHQS), 3-dehydroquinate dehydratase/shikimate 5-dehydrogenase (DHQ/SDH), shikimate kinase (SK), 5-enolpyruvylshikimate-3-phosphate synthase (EPSPS), and chorismate synthase (CS) [74]. In this study, one DHQ/SDH gene (*MD15G1335500*) was significantly up-regulated only in ‘ZC9-3’. Anthranilate serves as a precursor for tryptophan biosynthesis [75]; after drought stress, it was significantly up-regulated only in ‘ZC9-3’. The phenylpyruvate/4-hydroxyphenylpyruvate pathway and arogenate pathway (both via the reverse reaction order) have been identified to mediate the subsequent conversion of prephenate to phenylalanine and tyrosine. Although most microorganisms utilize only the first pathway, with a few exceptions [76], the major flux in plants is directed through the second pathway [77, 78]. In the first pathway, the final conversion of 4-hydroxyphenylpyruvate to tyrosine is catalyzed by TAT. This activity has been detected in mung bean, but the enzyme-encoding gene has not been isolated from mung bean or any other legume species [75]. In Arabidopsis, one of two known cytosolic TATs, AtTAT2, catalyzes the transamination of 4-hydroxyphenylpyruvate to tyrosine in *vitro* [79]. However, data in *planta* indicate that AtTAT2 contributes to tocopherol synthesis rather than to tyrosine biosynthesis under stress conditions [80]. In this study, one TAT gene (*MD03G1234800*) was significantly up-regulated in ‘Jizhen-2’ but significantly down-regulated in ‘ZC9-3’.

In plants, the saccharopine pathway is one of the main pathways mediating lysine catabolism, in which lysine is converted to α-aminoadipate by three enzymatic reactions catalyzed by lysine-ketoglutarate reductase (LKR), saccharopine dehydrogenase (SDH), and α-aminoadipate semialdehyde dehydrogenase (AASADH). The glutamate produced in the second step of this pathway can be metabolized by P5CS and pyrroline-5-carboxylate reductase (P5CR) to produce Pro [81]. In addition, α-aminoadipate appears to play a critical role in determining the degree of serotonin accumulation and the color of the rice endosperm/grain [82]. In our study, the lower increase in the content of L-α-aminoadipate and Pro under drought stress was less pronounced in ‘Jizhen-2’ than in ‘ZC9-3’, and L-glutamate and two P5CS genes (*MD07G1204500* and *MD12G1150700*) were significantly up-regulated only in ‘ZC9-3’. P5CS is a single bifunctional enzyme with two catalytic domains; glutamyl kinase (GK) and glutamyl phosphate reductase (GPR) [83]. Pro can also be synthesized from ornithine, which can be synthesized from glutamate through the sequential catalysis of *N*-acetylglutamate synthase (NAGS), *N*-acetylglutamate kinase (NAGK), NAGPR, *N*-acetylornithine aminotransferase (NAOAT), and *N*-acetylornithine deacetylase (NAOD) [84]. In our study, one NAGPR gene (*MD09G1171500*) was significantly up-regulated only in ‘ZC9-3’ under drought stress.

## Conclusion

Compared with ‘Jizhen-2’, ‘ZC9-3’ had a higher RWC and photosynthetic, antioxidant, and osmoregulation capacities under drought stress, indicating that the drought resistance of ‘ZC9-3’ is higher than that of ‘Jizhen-2’. Amino acids and derivatives are key metabolites of the two cultivars in response to drought stress, and the amino acid biosynthesis pathway is a key pathway underlying the drought resistance of apple rootstocks. A comprehensive metabolomic and transcriptomic analysis of the amino acid biosynthesis pathway revealed that 13 key metabolites contributed to the difference in drought resistance between ‘ZC9-3’ and ‘Jizhen-2’, including L-α-aminoadipate, L-homoserine, L-threonine, L-isoleucine, L-valine, L-leucine, (2*S*)-2-isopropylmalate, anthranilate, L-tryptophan, L-phenylalanine, L-tyrosine, L-glutamate, and L-Pro. In addition, 13 key genes positively regulate the response of ‘ZC9-3’ to drought stress, including one OASTL gene (*MD03G1184200*), two SAMS genes (*MD17G1165400* and *MD16G1138300*), one KARI gene (*MD17G1231900*), one DHAD gene (*MD04G1003600*), one IPMI gene (*MD13G1222900*), one BCAT gene (*MD15G1063000*), one PK gene (*MD13G1202600*), one DHQ/SDH gene (*MD15G1335500*), one TAT gene (*MD03G1234800*), one NAGPR gene (*MD09G1171500*), and two P5CS genes (*MD07G1204500* and *MD12G1150700*).

### Electronic supplementary material

Below is the link to the electronic supplementary material.

Supplementary Material 1

Supplementary Material 2

Supplementary Material 3. Additional file 1: Supplementary Figs. S1–S9

Supplementary Material 4

Supplementary Material 5

Supplementary Material 6

Supplementary Material 7

Supplementary Material 8

Supplementary Material 9

Supplementary Material 10

Supplementary Material 11

Supplementary Material 12

Supplementary Material 13

Supplementary Material 14

Supplementary Material 15

## Data Availability

Data supporting this study are included within the article and/or supporting materials.The datasets generated and analysed during the current study are available in the Sequence Read Archive (SRA) repository at the National Center for Biotechnology Information (NCBI) under the accession number PRJNA1066126.

## Abbreviations

ROS: Reactive oxygen species
MDA: Malondialdehyde
SOD: Superoxide dismutase
Pro: Proline
RWC: Leaf relative water content
Chl a: Chlorophyll *a*
Chl b: Chlorophyll *b*
Car: Carotenoids
Chl t: Total chlorophyll
Pn: Photosynthetic rate
Gs: Stomatal conductance
Ci: Intercellular CO_2_ concentration
Tr: Transpiration rate
WUE_I_: Instantaneous water use efficiency
HCA: Hierarchical cluster analysis
PCA: Principal component analysis
OPLS-DA: Orthogonal partial least squares-discriminant analysis
QC: Quality control
DAMs: Differentially accumulated metabolites
KEGG: Kyoto Encyclopedia of Genes and Genomes
DEGs: Differentially expressed genes
TFs: Transcription factors
PCCs: Pearson’s correlation coefficients
O2PLS: Two-way orthogonal partial least squares
