# Supplementary material for: Integrative physiological, metabolomic, and transcriptomic analysis reveals the drought responses of two apple rootstock cultivars
Source: BMC Plant Biol. 2024 Mar 26;24:219. doi: 10.1186/s12870-024-04902-2 (PMC10964572; doi:10.1186/s12870-024-04902-2)
Supplement: Supplementary file 3 — Supplementary Material 3. Additional file 1: Supplementary Figs. S1–S9 [file 12870_2024_4902_MOESM3_ESM.docx]

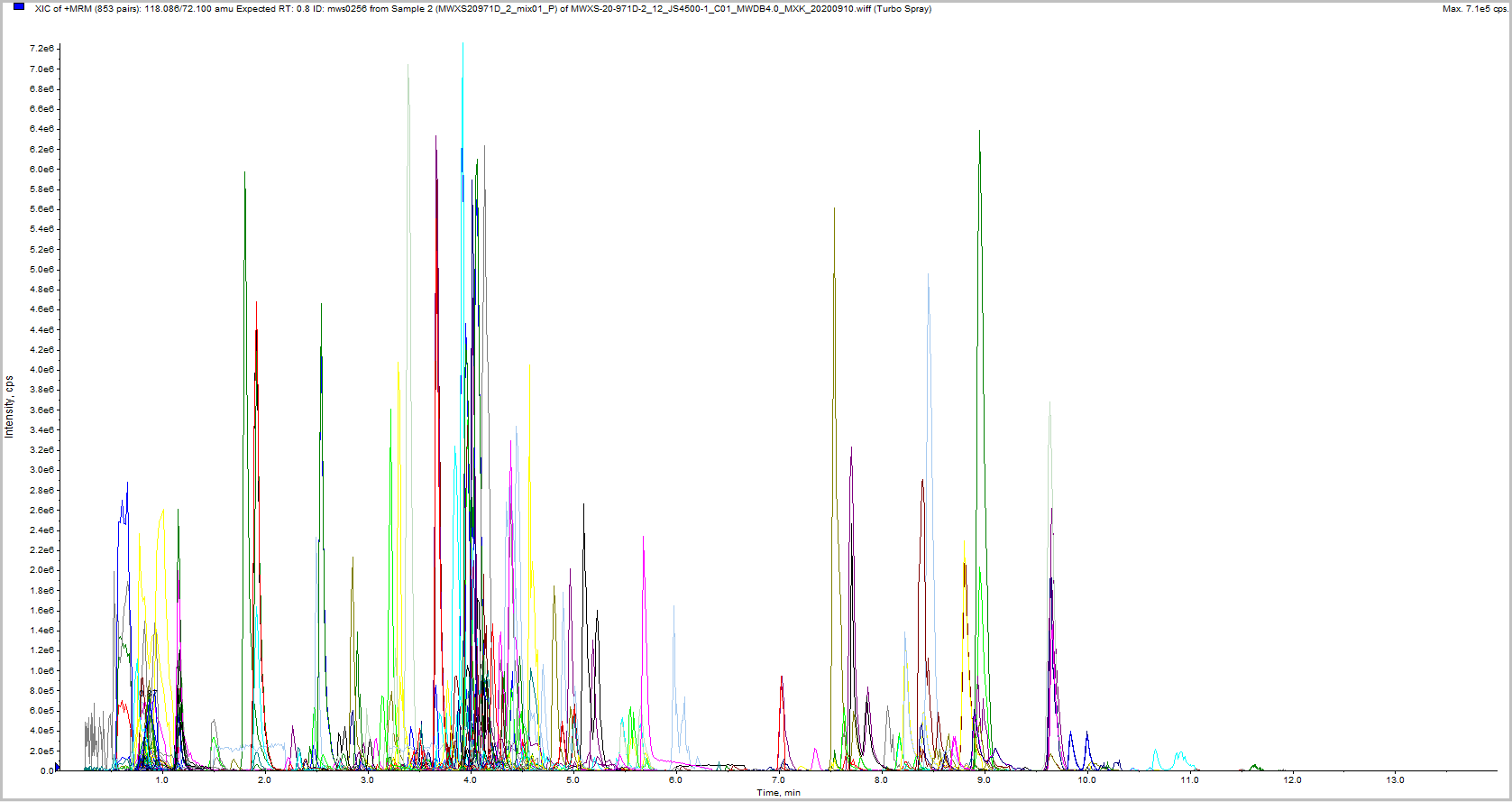


**A**


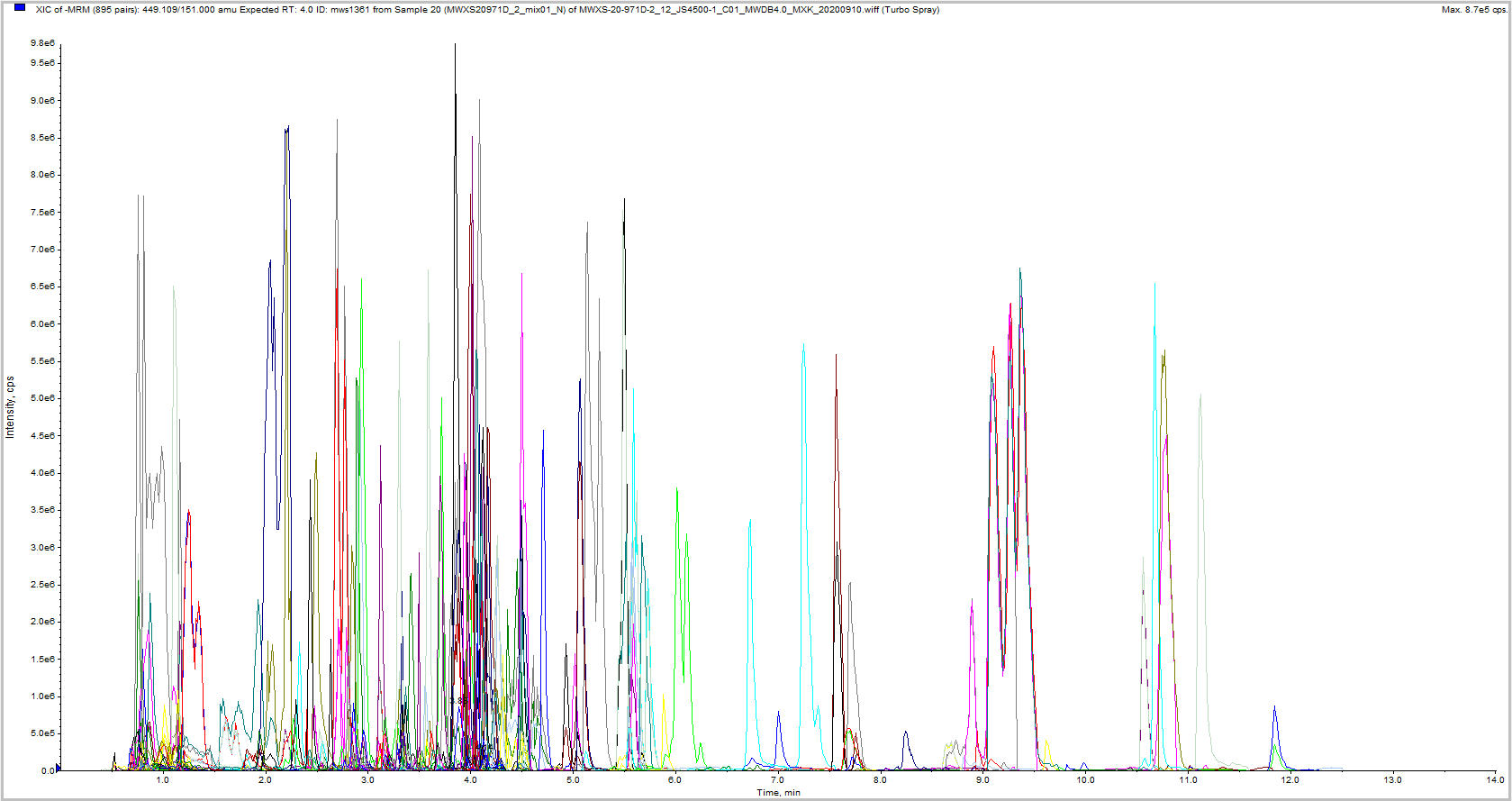


**B**

**Fig. S1** Multipeak map of MRM metabolite detection (ion current spectrum of multi-material extraction, XIC). (**A**), The detection in positive ion mode. (**B**), The detection in negative ion mode. The abscissa is the retention time of the metabolite detection (retention time, Rt). The ordinate is the ion current intensity of the ion detection (the intensity units are counts per second (cps)).


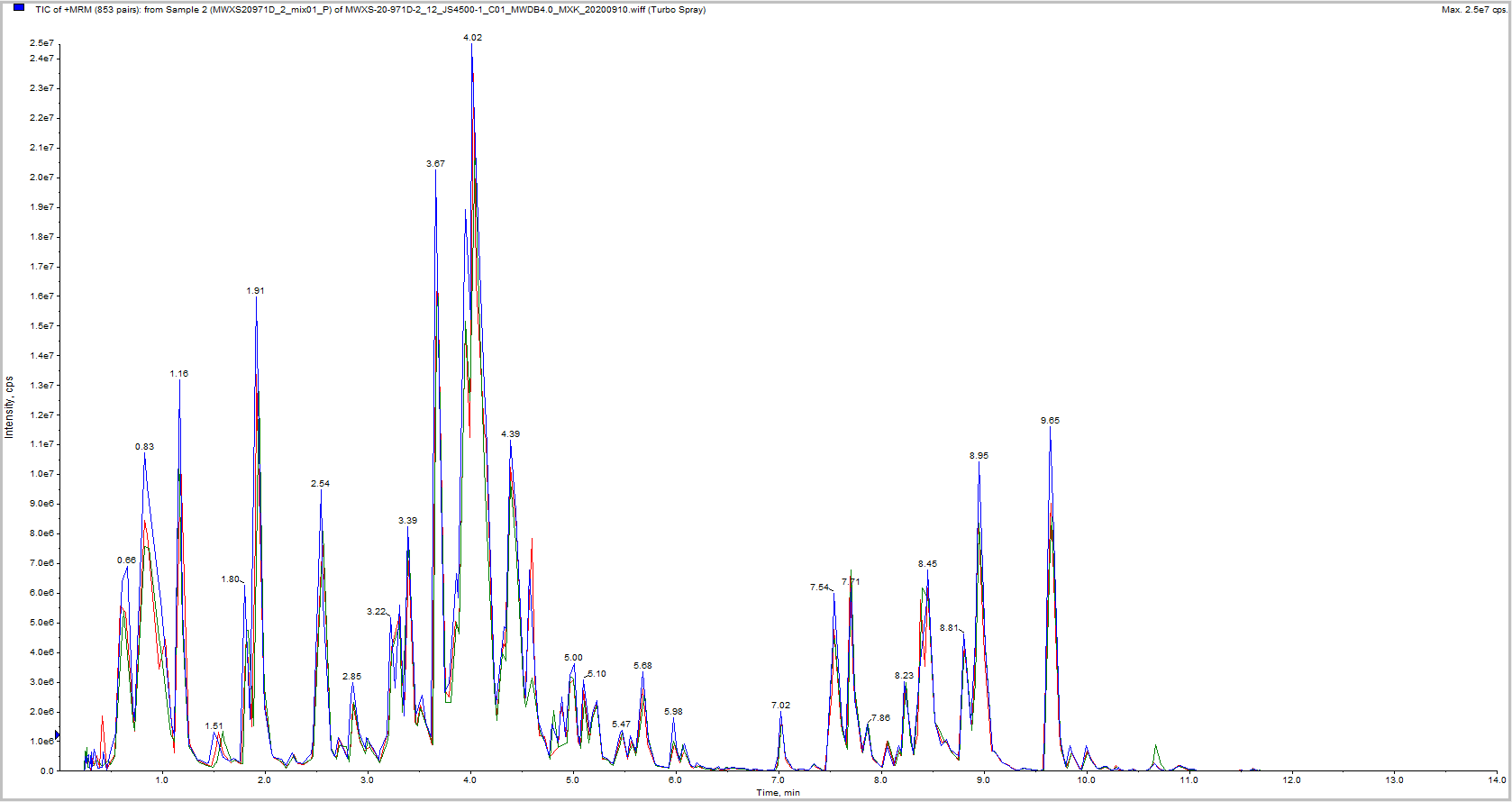


**A**

**B**


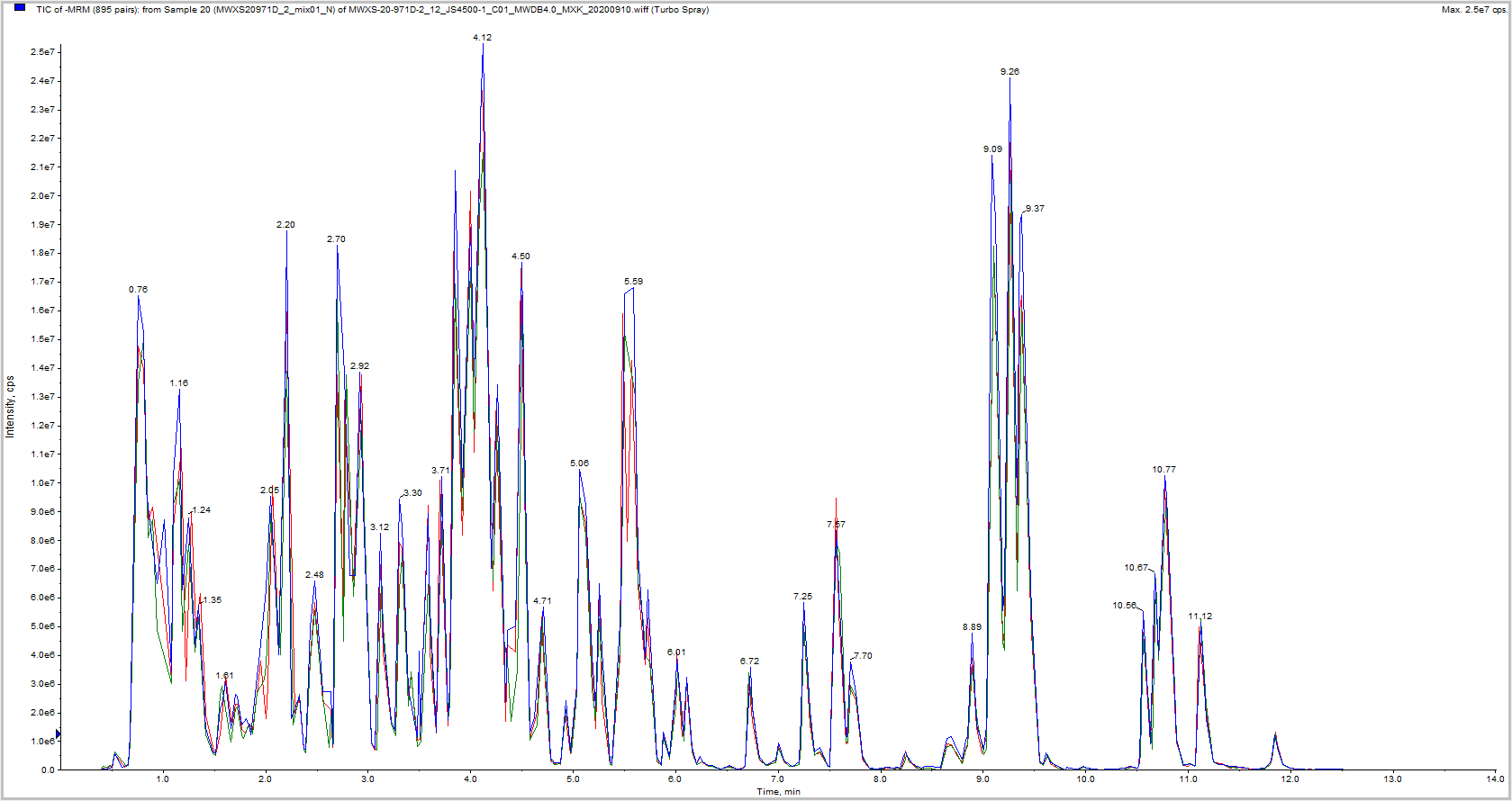


**Fig. S2** Total ions current (TIC) overlapping map of QC samples mass spectrometry (MS) results. (**A**), The detection in positive ion mode. (**B**), The detection in negative ion mode. The abscissa is the retention time of the metabolite detection (retention time, Rt). The ordinate is the ion current intensity of the ion detection (the intensity units are counts per second (cps)).


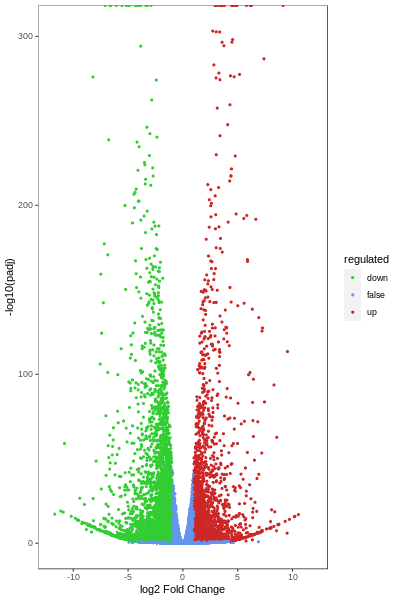

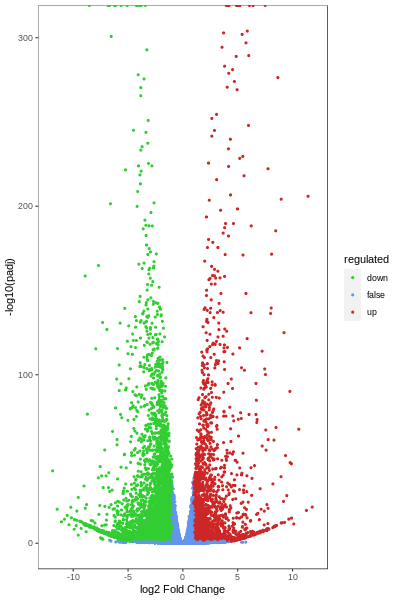


**A**

**B**

**Fig. S3** Volcano plot of differentially expressed genes (DEGs) in the Jizhen-2-CK vs. Jizhen-2-D and ZC9-3-CK vs. ZC9-3-D comparisons. (**A**), The number of DEGs in the Jizhen-2-CK vs. Jizhen-2-D comparison. (**B**), The number of DEGs in the ZC9-3-CK vs. ZC9-3-D comparison. Each point in the figure represents a DEG.


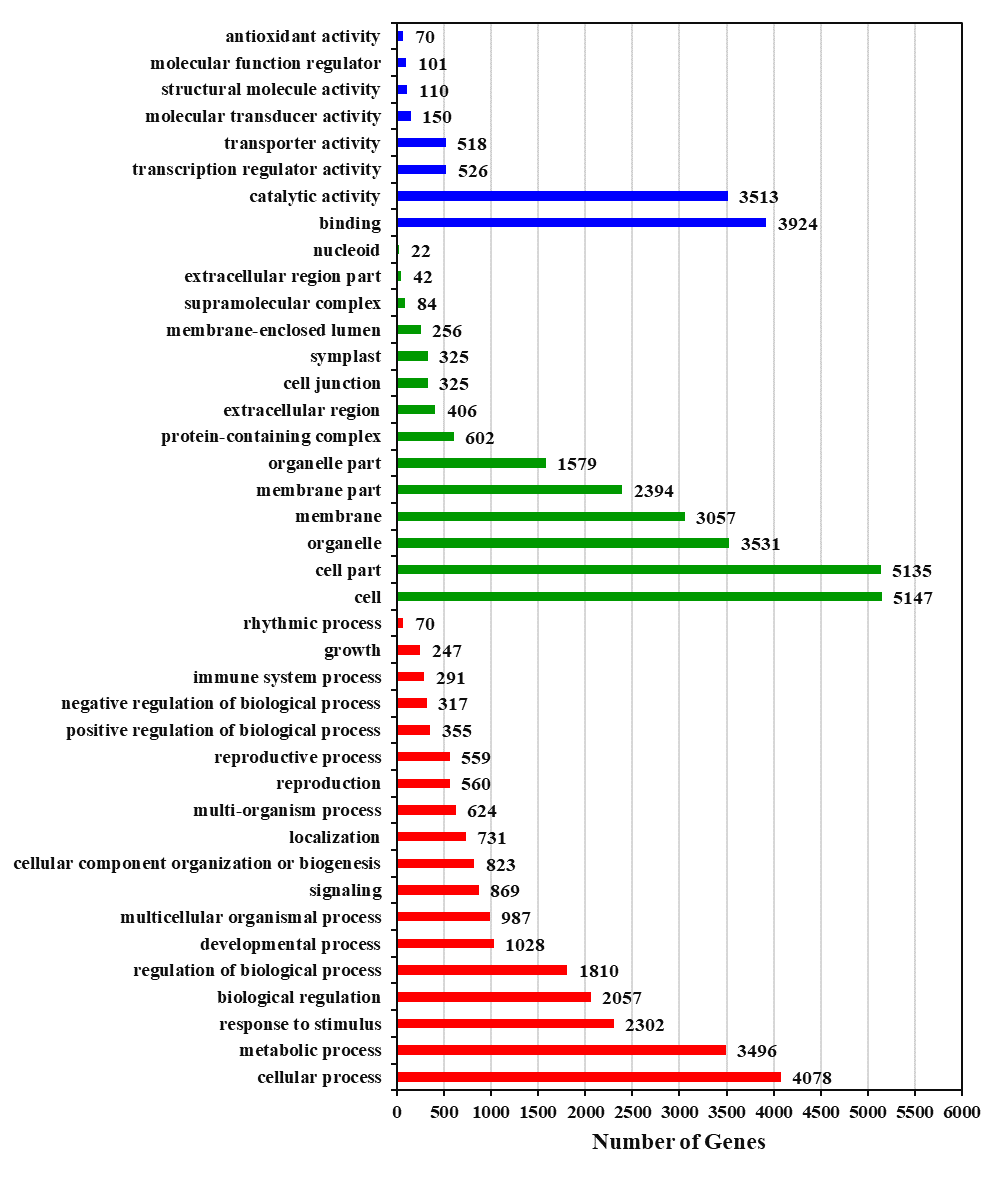

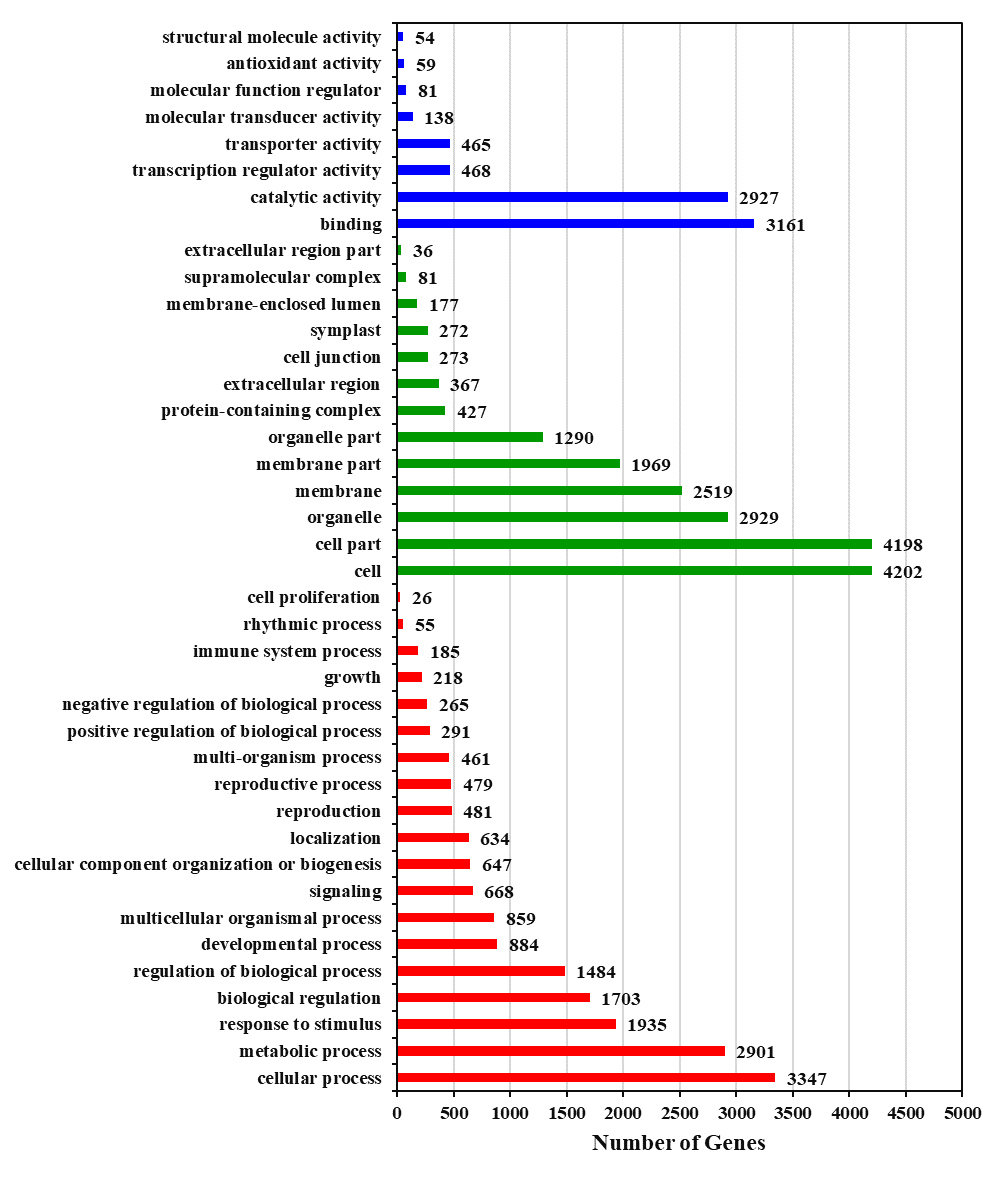


**Cellular component**

**Molecular function**

**Biological process**

**Cellular component**

**Molecular function**

**Biological process**

**A**

**B**

**Fig. S4** Gene Ontology **(**GO) enrichment analysis of DEGs in the Jizhen-2-CK vs. Jizhen-2-D and ZC9-3-CK vs. ZC9-3-D comparisons. (**A**), GO enrichment analysis of DEGs in the Jizhen-2-CK vs. Jizhen-2-D comparison. (**B**), GO enrichment analysis of DEGs in the ZC9-3-CK vs. ZC9-3-D comparison.

**
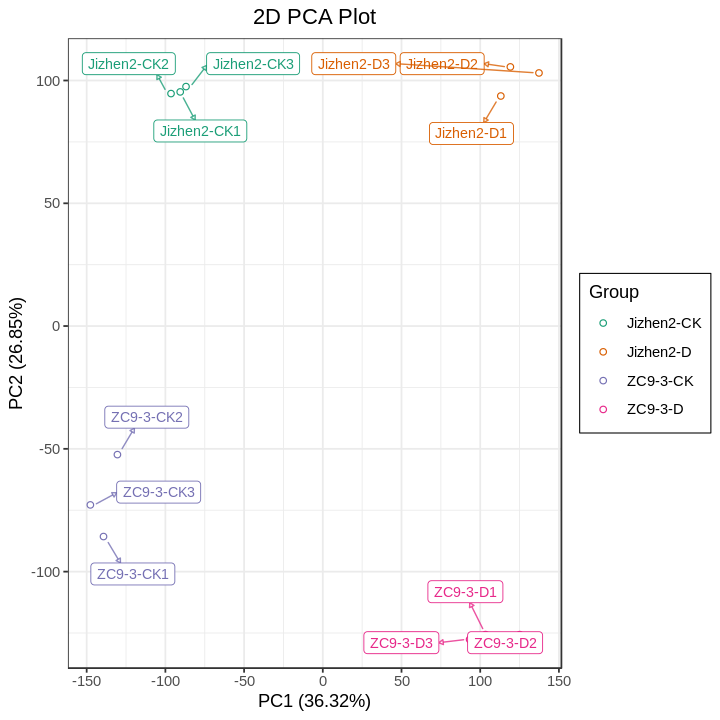
Fig. S5** Principal component analysis (PCA) of the transcriptomic data from Jizhen-2-CK, Jizhen-2-D, ZC9-3-CK, and ZC9-3-D samples.


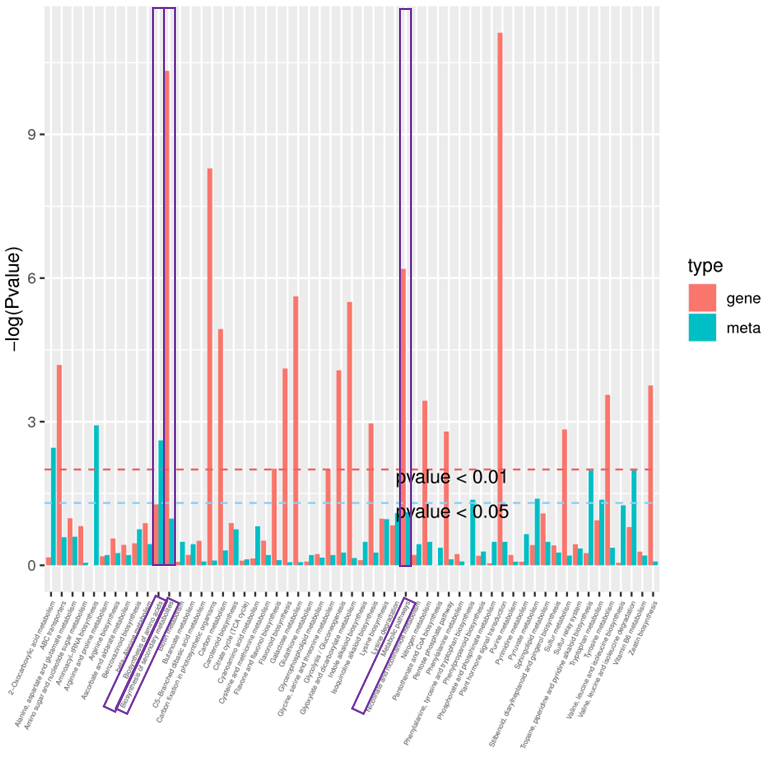


**A**

**B**


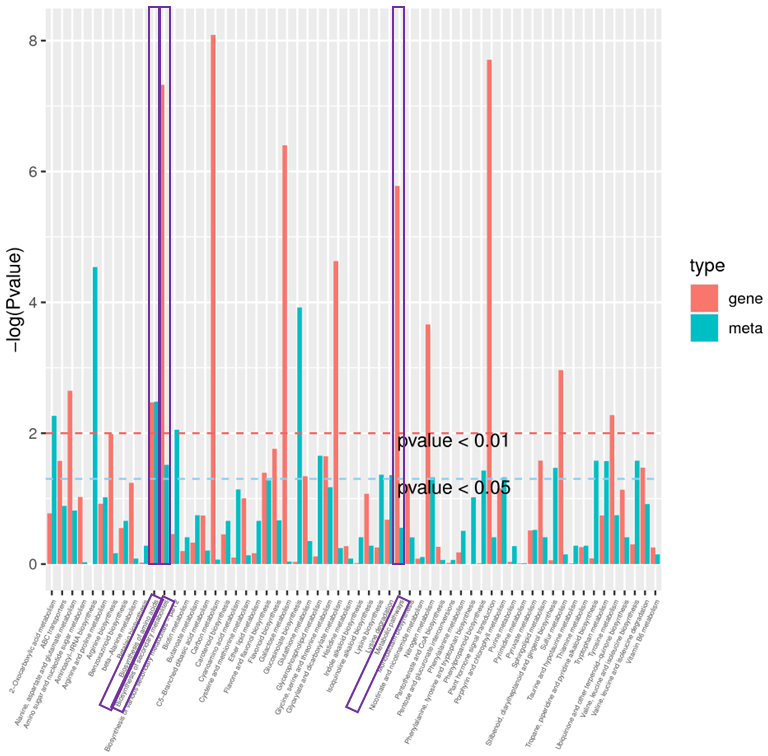


**Fig. S6** KEGG enrichment analysis of DAMs and DEGs in the Jizhen-2-CK vs. Jizhen-2-D and ZC9-3-CK vs. ZC9-3-D comparisons. (**A**), KEGG enrichment analysis of DAMs and DEGs in the Jizhen-2-CK vs. Jizhen-2-D comparison. (**B**), KEGG enrichment analysis of DAMs and DEGs in the ZC9-3-CK vs. ZC9-3-D comparison.

**Fig. S7** Nine-quadrant diagram of the difference multiples of the metabolites with Pearson’s correlation coefficient (PCC) > 0.8 in the Jizhen-2-CK vs. Jizhen-2-D and ZC9-3-CK vs. ZC9-3-D comparisons. (**A**), Nine-quadrant diagram of the Jizhen-2-CK vs. Jizhen-2-D comparison. (**B**), Nine-quadrant diagram of the ZC9-3-CK vs. ZC9-3-D comparison.


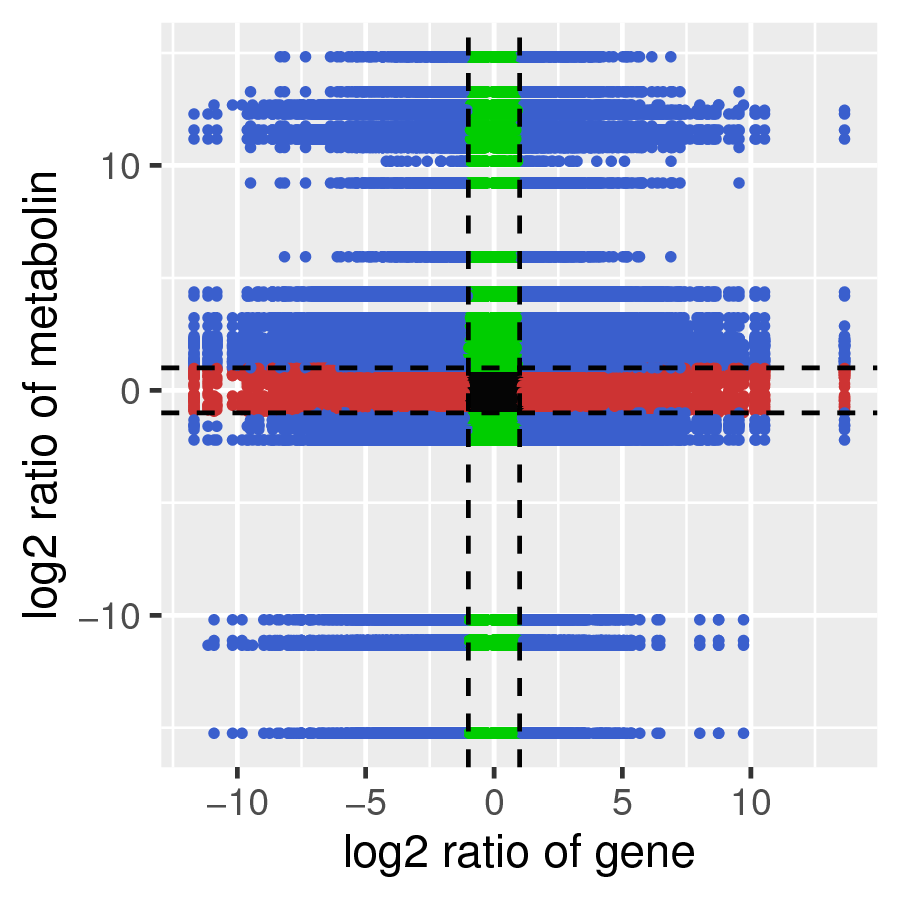


**A**


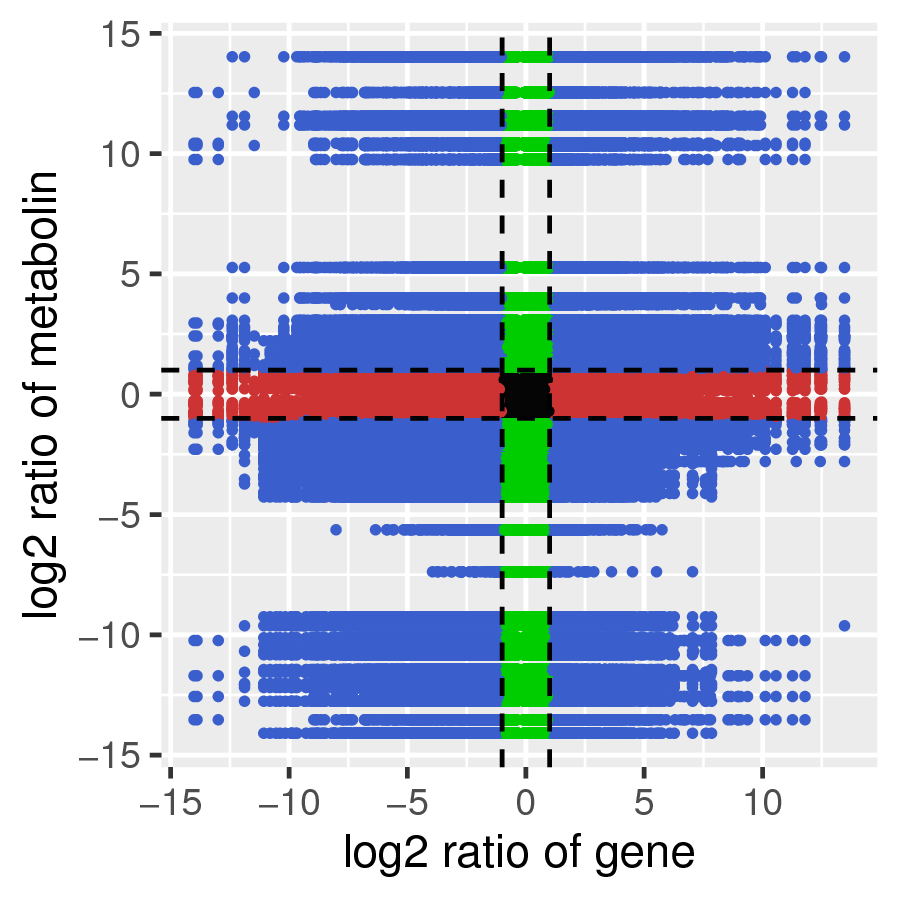


**B**

**A**

**B**


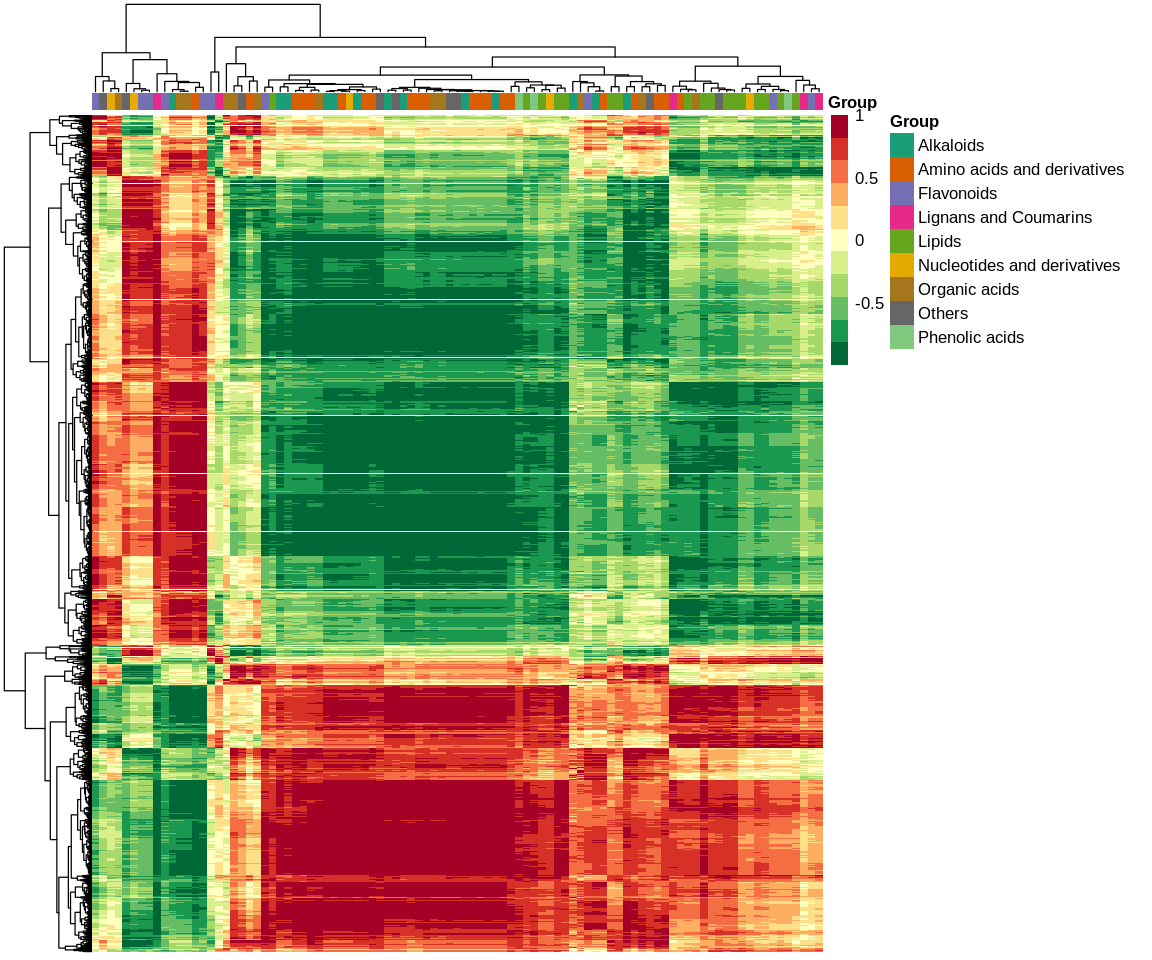

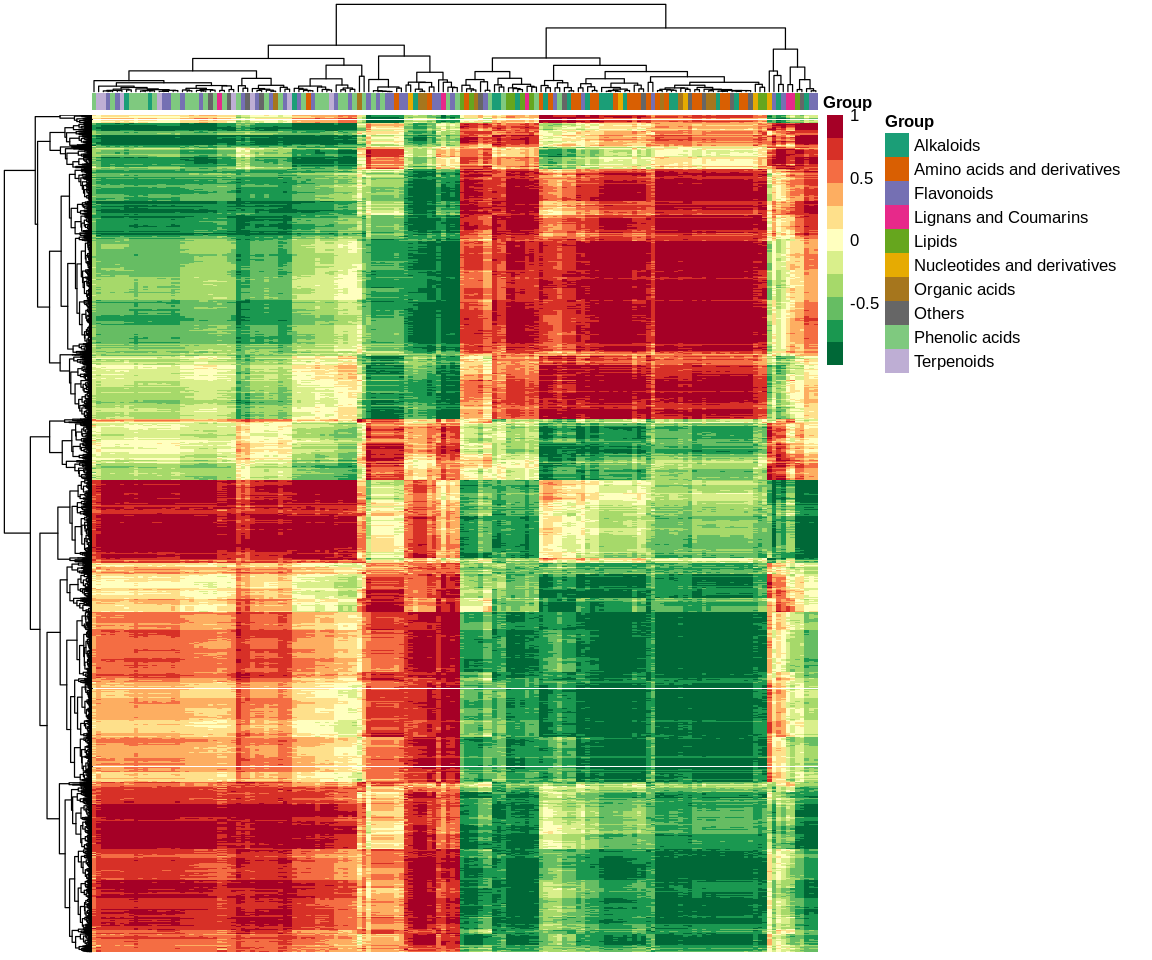


**Fig. S8** Differential correlation analysis (PCC >0.8) of the Jizhen-2-CK vs. Jizhen-2-D and ZC9-3-CK vs. ZC9-3-D comparisons. (**A**), Differential correlation analysis (PCC >0.8) of the Jizhen-2-CK vs. Jizhen-2-D comparison. (**B**), Differential correlation analysis (PCC >0.8) of the ZC9-3-CK vs. ZC9-3-D comparison.


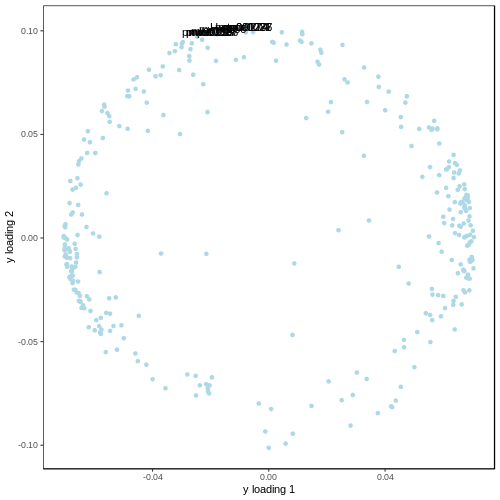


**Fig. S9** The 10 metabolites affected most significantly by the transcriptome.
